# Redox signaling through zinc activates the radiation response in *Deinococcus* bacteria

**DOI:** 10.1038/s41598-021-84026-x

**Published:** 2021-02-25

**Authors:** Romaric Magerand, Pascal Rey, Laurence Blanchard, Arjan de Groot

**Affiliations:** 1 Aix Marseille Univ, CEA, CNRS, BIAM, Molecular and Environmental Microbiology Team, 13108 Saint Paul-Lez-Durance, France; 2 Aix Marseille Univ, CEA, CNRS, BIAM, Plant Protective Proteins Team, 13108 Saint Paul-Lez-Durance, France

**Keywords:** Gene regulation, Prokaryote, Metals, Proteases, Structural biology, DNA damage and repair, Bacteria, Environmental microbiology, Microbial genetics

## Abstract

*Deinococcus* bacteria are extremely resistant to radiation and other DNA damage- and oxidative stress-generating conditions. An efficient SOS-independent response mechanism inducing expression of several DNA repair genes is essential for this resistance, and is controlled by metalloprotease IrrE that cleaves and inactivates transcriptional repressor DdrO. Here, we identify the molecular signaling mechanism that triggers DdrO cleavage. We show that reactive oxygen species (ROS) stimulate the zinc-dependent metalloprotease activity of IrrE in *Deinococcus*. Sudden exposure of *Deinococcus* to zinc excess also rapidly induces DdrO cleavage, but is not accompanied by ROS production and DNA damage. Further, oxidative treatment leads to an increase of intracellular free zinc, indicating that IrrE activity is very likely stimulated directly by elevated levels of available zinc ions. We conclude that radiation and oxidative stress induce changes in redox homeostasis that result in IrrE activation by zinc in *Deinococcus*. We propose that a part of the zinc pool coordinated with cysteine thiolates is released due to their oxidation. Predicted regulation systems involving IrrE- and DdrO-like proteins are present in many bacteria, including pathogens, suggesting that such a redox signaling pathway including zinc as a second messenger is widespread and participates in various stress responses.

## Introduction

For all organisms it is important to respond efficiently to changes in environmental constraints leading to stress conditions within cells. In case of DNA damage-generating stress, many bacteria use the SOS response to induce expression of DNA repair genes. The molecular signal inducing the SOS response is the presence of single-stranded DNA^1^. RecA filaments formed on single-stranded DNA stimulate self-cleavage and inactivation of LexA, the repressor of the SOS response. Not all bacteria use the SOS response to induce DNA repair genes^2^. In *Deinococcus* bacteria, induction of *recA* and other DNA repair genes occurs in a RecA/LexA-independent manner and in the present work we aim to identify the intracellular molecular signal that induces this SOS-independent response.

*Deinococcus* species are famous for their extreme tolerance to DNA damage- and oxidative stress-generating conditions such as radiation and desiccation, and for their capacity to repair massive DNA damage^3–6^. This extreme tolerance results from a combination of multiple factors and mechanisms^7^, including limitation of oxidative protein damage^8–10^ that has been correlated with a high intracellular Mn^2+^/Fe^2+^ ratio (e.g. 0.24 in *Deinococcus radiodurans* versus < 0.01 in radiation-sensitive *Escherichia coli*)^11^ and the accumulation of small antioxidant Mn^2+^-containing complexes (with intracellular Mn^2+^ concentrations ranging from 0.2 to 2 mM, 70% of which not bound to proteins)^12^. An efficient radiation/desiccation response (RDR) mechanism for induced expression of a set of genes forming the RDR regulon is also essential for radiation tolerance^13–16^. The predicted RDR regulon in different *Deinococcus* species consists of about 20 genes, including classical DNA repair genes (e.g. *recA*, *uvrB*) and novel genes more specific to *Deinococcus* (e.g. *ddrB*, *ddrO*)^17^. We previously demonstrated that RDR regulon expression is controlled by two proteins highly conserved in *Deinococcus* species: IrrE and DdrO^14^.

IrrE, also called PprI, is a COG2856 domain-containing metalloprotease that induces expression of the RDR genes by cleaving and inactivating DdrO, the transcriptional repressor of the RDR regulon^14,17–20^. DdrO has a helix-turn-helix (HTH) motif-containing DNA-binding domain of the XRE family^19^. The HTH_XRE-type domain is present in many transcriptional regulators and has been named after the PBSX repressor protein Xre from the defective *Bacillus* prophage PBSX^21^. DdrO cleavage has been demonstrated when IrrE and DdrO are co-expressed from plasmids in *E. coli* without exogenous stress, and in vitro with both proteins purified from *E. coli*. In *Deinococcus* itself, however, the cleavage is somehow induced when bacteria are exposed to radiation^14^. The N-terminal domain of IrrE is structurally similar to zinc metalloprotease thermolysin^22^, and includes the active site/metal ion binding motif HEXXH that is essential for protease activity^14^. No metal ion was observed in the crystal structure of IrrE, but briefly soaking apo-IrrE crystals in a Zn^2+^-containing solution resulted in binding of Zn^2+^ in the expected site, accompanied by re-orientation of the side chains of two active site residues towards the Zn^2+^ ion^22^. Addition of Zn^2+^ to metal-free IrrE was sufficient to restore DdrO cleavage in vitro^17^. The in vitro protease activity of IrrE could also be restored after addition of Mn^2+^ or Fe^2+^ ions to metal-free enzyme^17^. Such in vitro re-activation not only by Zn^2+^ but also by some other metals is commonly found for zinc metalloproteases, including thermolysin^23,24^. Besides its protease domain, the crystal structure of IrrE revealed an additional domain with structural similarity to GAF domains^22^, which may have a role in signaling or in protein–protein interactions^25,26^. Interestingly, predicted but largely uncharacterized COG2856/XRE protein pairs have been identified in many other bacterial genera, including pathogens and bacteria used in biotechnological industry^14,19,27–29^, suggesting that stress response mechanisms involving such protein pairs are more widespread than currently recognized.

The molecular signaling mechanism by which radiation triggers cleavage of DdrO by the constitutively expressed IrrE in *Deinococcus* is unknown. Exposure of the cells to radiation or desiccation causes redox imbalance through formation of reactive oxygen species (ROS)^6^. ROS can react with and damage any molecule in the cell, including DNA. In turn, DNA damage can trigger ROS production^30^. Besides causing damage, ROS and other reactive species may also activate proteins through oxidative post-translational modification of cysteine residues, as exemplified by chaperone Hsp33 and transcription factor OxyR in *E. coli* and several other bacteria^31,32^. Here, we investigated if ROS are involved in IrrE activation, either by direct redox modification of IrrE or indirectly by generating another molecular signal acting downstream the stress-induced ROS and redox imbalance. We show that IrrE is a zinc-dependent enzyme in vivo in *Deinococcus*, that induction of IrrE protease activity still occurs when IrrE lacks its GAF-like domain, and that IrrE is activated directly by increased intracellular levels of free zinc ions following a zinc shock. Moreover, an increase in free zinc and IrrE protease activation is also observed after oxidative treatment of the cells. We propose that induction of the RDR regulon by radiation and oxidative stress involves a zinc signal generated by impaired redox homeostasis. Such role for zinc as a second messenger in regulation of cellular enzyme activities has been described previously for eukaryotes^33–35^, but to our knowledge this is the first time for prokaryotes.

## Results

### DdrO cleavage in *Deinococcus* is stimulated by oxidative treatments

**Figure 1:**
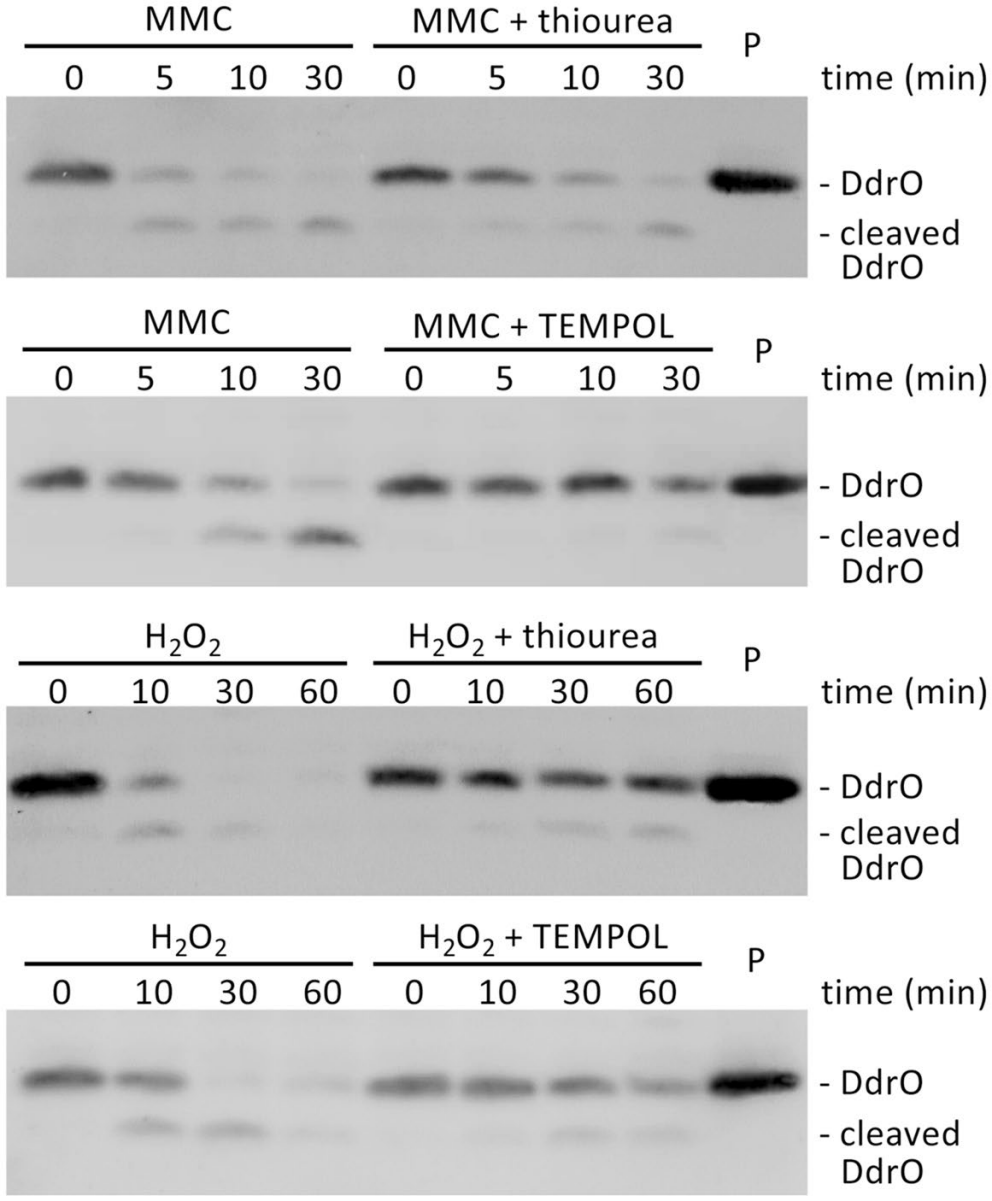
Mitomycin C and H_2_O_2_ induce DdrO cleavage in *D. deserti*. Wild-type cells were exposed to MMC (0.5 μg/ml) or H_2_O_2_ (10 mM) for the indicated time in the absence or presence of the antioxidants thiourea (150 mM) or TEMPOL (10 mM), and DdrO cleavage was analyzed by immunoblotting. P, 20 ng purified DdrO (129 aa, 14.7 kDa). Each cropped blot corresponds to a single independent blot; the four uncropped blots are shown in Supplementary Fig. S9.

Cleavage of RDR regulon repressor DdrO (129 aa) by metalloprotease IrrE (281 aa) in *D. deserti* has been observed after exposure of the cells to gamma or UV radiation^14^. The cleavage occurs between L106 and R107 of DdrO. Here we show that this cleavage is also induced after a short exposure to mitomycin C (MMC) (Fig. 1; Supplementary Fig. S1). MMC is known in particular as an alkylating agent that generates interstrand DNA cross-links, but the metabolism of this quinone-containing antibiotic in the cell also results in oxidative damage to other macromolecules via the generation of various ROS^6,36–38^. The antioxidant compound thiourea scavenges hydroxyl and superoxide radicals and hydrogen peroxide (H_2_O_2_)^39,40^, and has been found to inhibit the effects of the MMC-induced ROS production in *E. coli*^36^. Here we observed that the MMC-induced DdrO cleavage in *D. deserti* was decreased in the presence of thiourea (Fig. 1; Supplementary Fig. S1), suggesting the involvement of MMC-induced ROS in DdrO cleavage stimulation. Another antioxidant, TEMPOL, which catalyzes superoxide dismutation, facilitates catalase-like metabolism of H_2_O_2_ and limits hydroxyl radical formation^41–43^, also inhibited DdrO cleavage in cells exposed to MMC (Fig. 1). When the cells were directly exposed to H_2_O_2_ for 10 min, DdrO cleavage was also efficiently induced, which was inhibited by thiourea and TEMPOL (Fig. 1), further confirming the participation of redox signaling in IrrE activation.

We first presumed that stress-generated ROS directly oxidize IrrE. The sulfur-containing amino acids methionine (Met) and cysteine (Cys) are particularly sensitive to oxidation, and fulfill a key role in redox signaling. Thus for some proteins oxidation of these residues leads to their activation (e.g. HypT, OxyR, Hsp33)^44^. Met and Cys residues highly conserved in deinococcal IrrE proteins were mutated in *D. deserti* IrrE. None of the mutations affected radiation resistance and DdrO cleavage in *D. deserti* (Supplementary Fig. S2), excluding a role of these residues in IrrE activation upon stress exposure. This is in line with the previously reported in vitro cleavage of DdrO by IrrE, using purified proteins for which any modification (e.g. oxidized residues) was not detected by mass spectrometry^14,17^. Together, these data suggest that ROS stimulate IrrE activity in an indirect manner.

### IrrE is a zinc-dependent metalloprotease in *Deinococcus*

**Figure 2:**
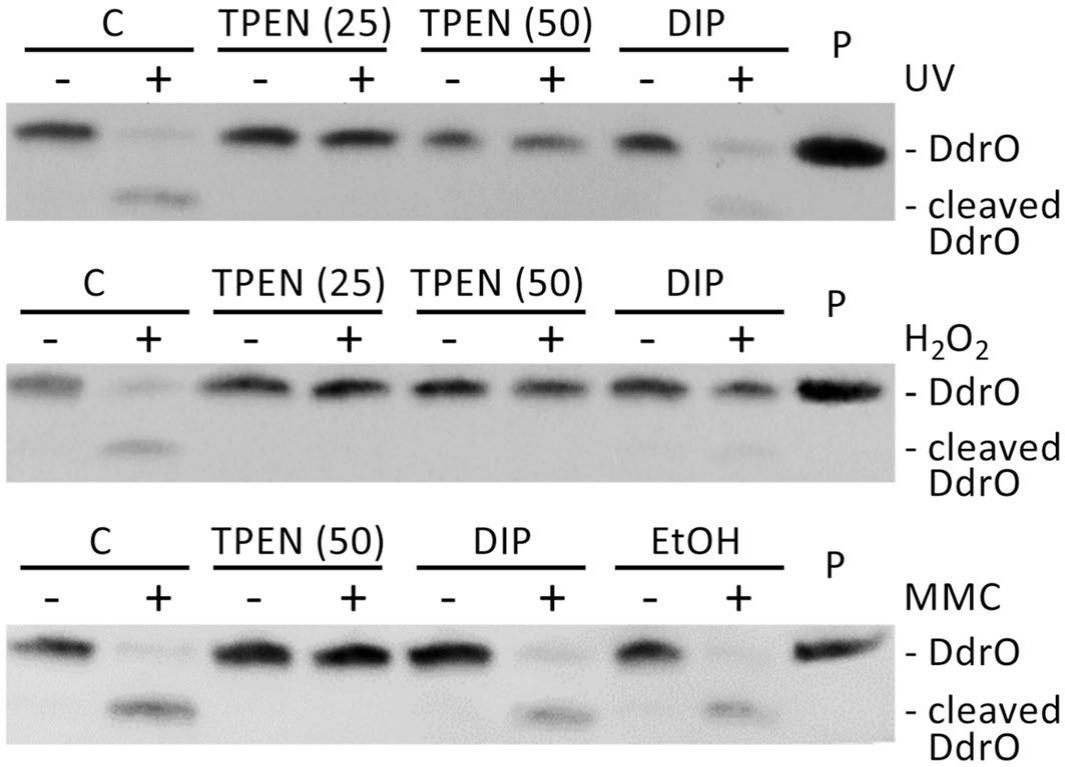
Stress-induced DdrO cleavage in *D. deserti* requires zinc ions. *D. deserti* strain RD19 (wild type) was exposed or not to UV (250 J/m^2^), H_2_O_2_ (10 mM for 10 min) or MMC (1 μg/ml for 10 min) in the presence of the Zn^2+^ chelator TPEN (25 or 50 μM) or the Fe^2+^ chelator DIP (500 μM), and DdrO cleavage was analyzed by immunoblotting. Because TPEN was dissolved in ethanol (EtOH), a control with EtOH (0.5% final concentration) was included. C, control without metal chelator. P, 20 ng purified DdrO. Each cropped blot corresponds to a single independent blot; the three uncropped blots are shown in Supplementary Fig. S9.

DdrO cleavage by IrrE is not only stimulated by radiation and other stresses that induce ROS production, but also rapidly (within 5–10 min) and efficiently after exposure of *Deinococcus* cultures to an excess of zinc ions, but not of other metal ions including Mn^2+^ and Fe^2+^^17^, as confirmed here (Supplementary Fig. S1). The stimulation of IrrE protease activity by the zinc shock might be direct, if IrrE activity indeed requires Zn^2+^ as co-factor in vivo, or indirect, if the zinc shock, in common with radiation, causes ROS formation and DNA damage. To better understand the zinc shock-induced activation mechanism, we first investigated the metal dependency of the IrrE activity in vivo by applying different treatments in the presence of TPEN or dipyridyl, which are membrane-permeable chelators with high affinity for zinc (Zn^2+^) and iron (Fe^2+^) ions, respectively. Dipyridyl (DIP) was included in these tests because of oxidative stress through ROS formation often involves Fe^2+^. DIP (500 μM) inhibited DdrO cleavage only in H_2_O_2_-exposed cells (Fig. 2; Supplementary Fig. S3), showing that the observed cleavage stimulated by H_2_O_2_, but not by UV or MMC, requires Fe^2+^, most likely via the Fenton reaction where H_2_O_2_ reacts with Fe^2+^ generating oxygen-radical species. TPEN (25 to 50 μM) totally blocked DdrO cleavage in *D. deserti* and *D. radiodurans* exposed to each of the applied treatments (Fig. 2; Supplementary Fig. S3). Additional experiments showed that adding 10 μM TPEN to the culture was sufficient to inhibit stress-induced DdrO cleavage (Supplementary Fig. S3). It has been suggested that Mn^2+^ might be the co-factor required for IrrE activity in vivo in *Deinococcus*^20^. However, the applied TPEN concentrations are low compared to the near-millimolar concentration of Mn^2+^ in *Deinococcus*, and the affinity of TPEN for Zn^2+^ (2.6 × 10^−16^ M) is much higher than for Mn^2+^ (5.4 × 10^−11^ M)^45^. Together, the data strongly support that the in vivo protease activity of IrrE is dependent on zinc ions.

### The zinc shock-induced cleavage is not accompanied by ROS production and DNA damage

**Figure 3:**
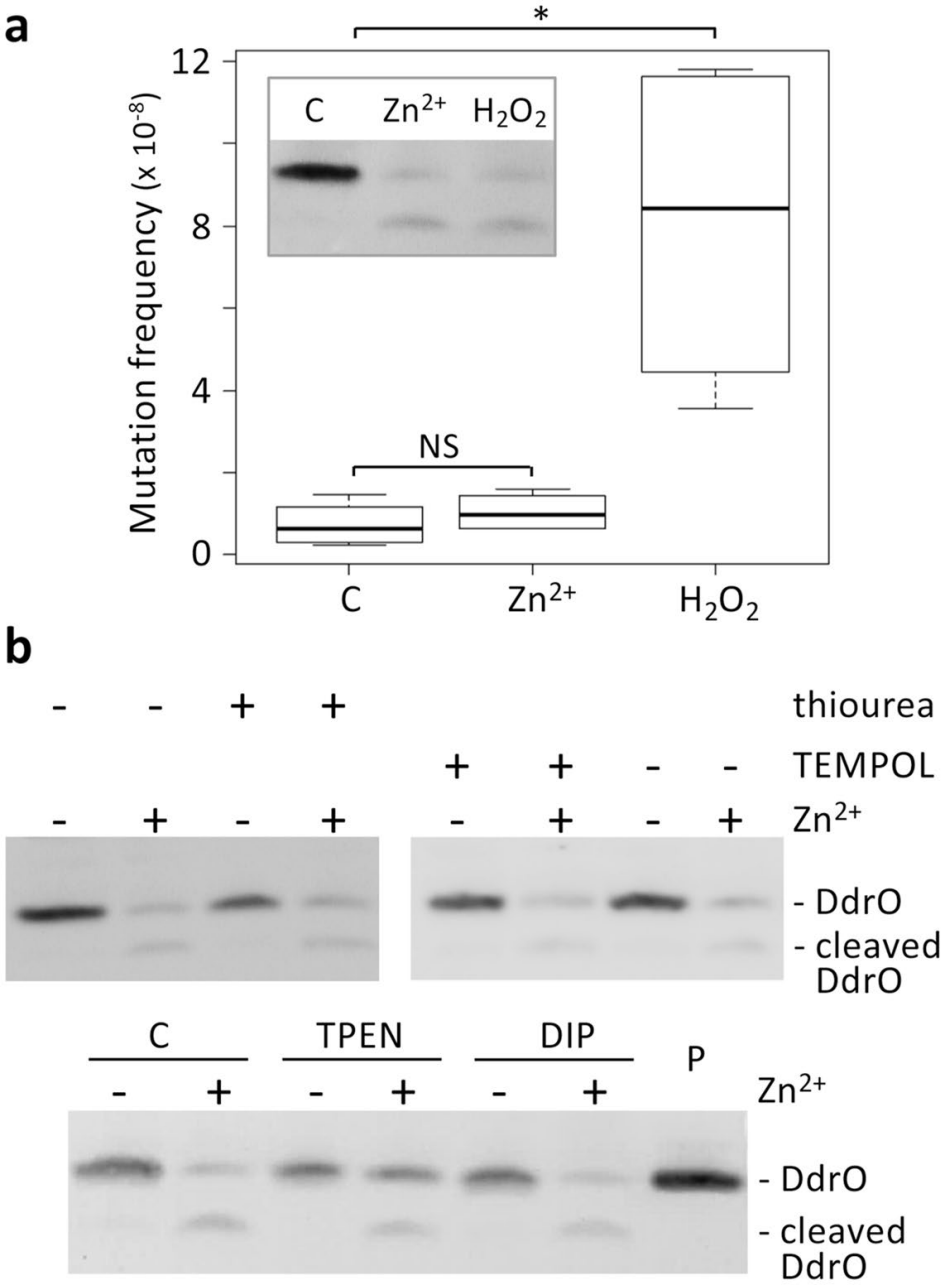
The zinc shock activates IrrE without generating ROS and DNA damage. (**a**) Induced mutagenesis experiments showing that the zinc shock-induced DdrO cleavage does not occur indirectly via DNA damage/oxidative stress. In contrast to the H_2_O_2_ exposure (10 mM for 10 min), the zinc shock (250 μM for 10 min) does not lead to an increase in DNA mutations, whereas both conditions induce DdrO cleavage (inlay). C, untreated control cells. A two-sided Mann–Whitney U test was performed on the data, and an asterisk indicates a *p* value < 0.05. NS, not significant. In box plots: center line, median; box limits, upper and lower quartiles; whiskers, 1.5 × interquartile range. (**b**) Western blots showing that DdrO cleavage induced by the zinc shock (250 μM for 10 min) is neither inhibited by antioxidants thiourea (150 mM) and TEMPOL (10 mM) nor by the Fe^2+^ chelator DIP (500 μM). The Zn^2+^ chelator TPEN (50 μM) partially inhibited the zinc shock-induced cleavage. C, control cells without metal chelator. P, 20 ng purified DdrO. Each cropped blot corresponds to a single independent blot; the four uncropped blots are shown in Supplementary Fig. S9.

Next, we analyzed whether the zinc shock-induced cleavage occurs indirectly via generation of ROS and/or DNA damage. We have previously shown that exposure of *D. deserti* to radiation results in an increase of point mutations as detected by an increased number of rifampicin resistant mutants^46^. Such mutations can arise through direct DNA damage or after incorporation of oxidized nucleotides. Efficient cleavage of DdrO was observed when *D. deserti* was exposed to either zinc shock or H_2_O_2_ treatment. However, in contrast to H_2_O_2_ exposure, zinc shock was not accompanied by induced mutagenesis (Fig. 3a). In addition, ROS were not detected following the zinc shock when a fluorescent ROS detector was used (Supplementary Fig. S4). These results indicate that DdrO cleavage by IrrE after the zinc shock is induced directly by the sudden increase of the Zn^2+^ concentration, and not indirectly via the generation of ROS and DNA damage. An increase in intracellular free Zn^2+^ following zinc shock was indeed observed using Fluozin-3 AM, a membrane-permeable molecule that after de-esterification in the cell can detect Zn^2+^ (Supplementary Fig. S5). The direct activation by Zn^2+^ is further supported by the observation that the antioxidants thiourea and TEMPOL did not inhibit the zinc-induced DdrO cleavage (Fig. 3b). Moreover, also dipyridyl did not inhibit the zinc-induced cleavage, showing that this induction did not occur indirectly via a step that involves Fe^2+^ (Fig. 3b).

**Figure 4:**
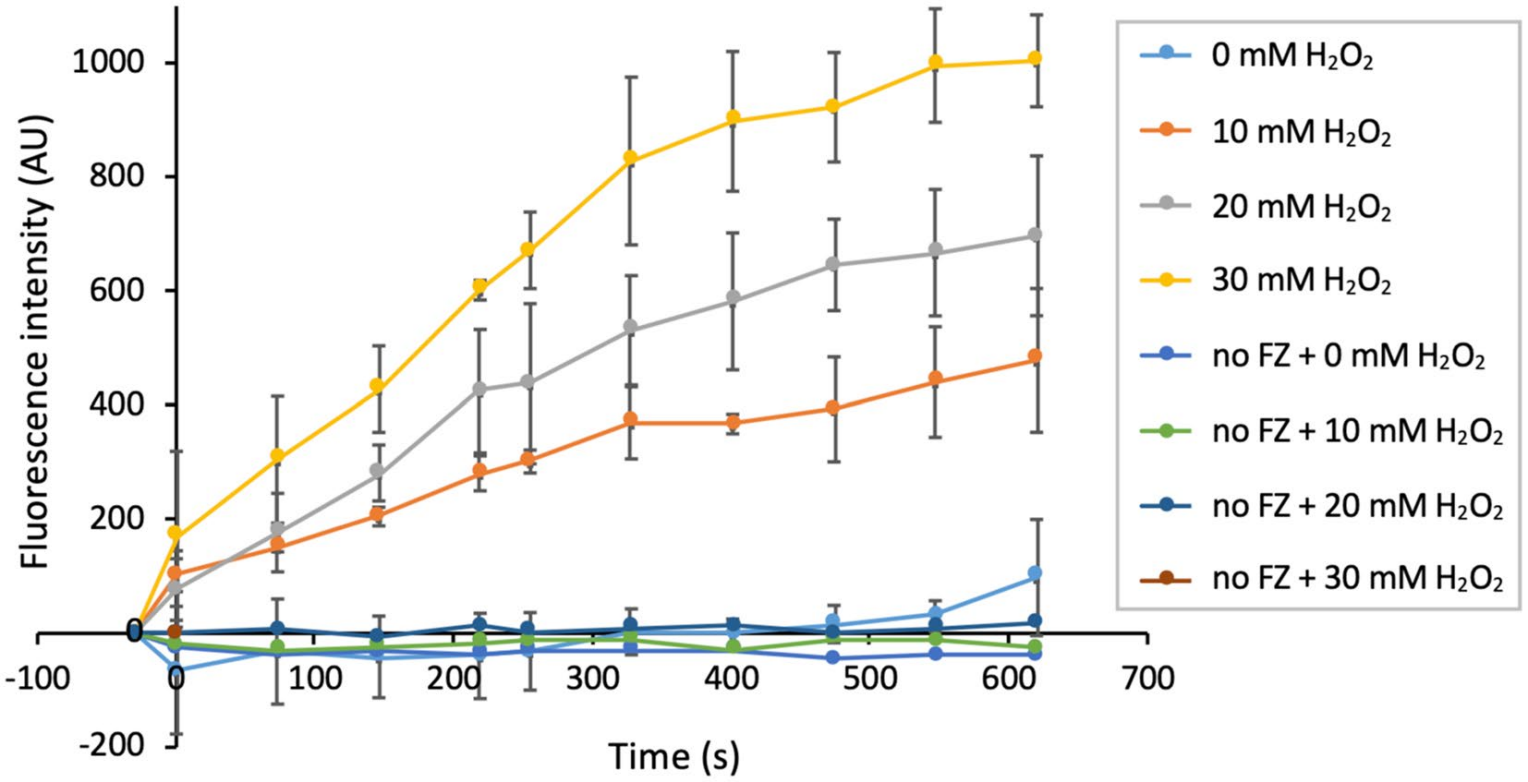
Detection of free intracellular zinc in *D. deserti* after H_2_O_2_ exposure. After de-esterification of FluoZin-3 AM in the cells, increase of free intracellular zinc was measured after addition of H_2_O_2_. The values are corrected for the fluorescence observed after the incubation for 60 min with FluoZin-3 AM but before H_2_O_2_ treatment. This observed fluorescence indicates that FluoZin-3 chelates intracellular Zn^2+^ already before treatment. Error bars are standard deviation of three independent experiments (biological replicates). no FZ, cells incubated without FluoZin-3 AM.

The direct stimulation of IrrE-mediated DdrO cleavage by zinc ions suggests that radiation/redox-induced cleavage may also involve an increase in free zinc ions. A ROS-induced increase in intracellular free zinc ions is strongly supported by several previous studies. Indeed, many oxidants and other reactive species can cause rapid release of zinc ions from redox-sensitive cysteine-containing zinc sites such as zinc fingers and related structures in proteins, which allows novel interactions between zinc ions and other proteins^31,33,34,47–49^. Therefore, we hypothesized that oxidative treatments leading to DdrO cleavage, such as exposure to H_2_O_2_, could modify the free zinc ion content in *Deinococcus*. Using Fluozin-3 AM we showed that H_2_O_2_ concentrations in the range of 10 to 30 mM indeed lead to substantial increases in the level of free zinc ions (Fig. 4). These data strongly support a role for zinc in oxidative-stress-induced activation of IrrE.

### Identification of *Deinococcus deserti* proteins with zinc/cysteine sites

This section describes possible sources of ROS-induced zinc mobilization in *D. deserti*. In addition to zinc/cysteine (Zn/Cys) sites in proteins, zinc ions can be bound by bacillithiol (BSH), a low-molecular-weight thiol produced by *Deinococcus* and several other bacteria, with oxidation of BSH (to BSSB) causing release of the zinc^50^. The zinc bound to BSH could thus be an important source for IrrE in oxidative conditions. However, with respect to DdrO cleavage upon stress and DdrO re-accumulation during recovery, no difference was observed between wild-type and BSH biosynthesis deficient mutant strains of *D. deserti* (Supplementary Fig. S6), showing that BSH is not crucial for regulating IrrE activity.

Release of zinc ions from Zn/Cys sites after oxidation or other modifications of the Zn^2+^-coordinating thiolates has been reported for several bacterial proteins^49,51^. Numerous proteins that bind zinc through Cys residues, or through a combination of Cys and histidine (His) residues, have been described or predicted. The zinc-binding residues are generally present as two pairs of closely spaced Cys/His (e.g. the most frequently found CXXC motif, but also others such as CXC, CXXXC and CXXH) separated by a longer spacer within the protein sequence^35,52^. To find *D. deserti* proteins that could be potential sources of oxidative stress-induced increase of intracellular free zinc, we searched its genome for proteins containing Zn/Cys sites. We found that it encodes homologs of at least 62 proteins harboring Zn/Cys sites (Supplementary Table S1), with a large fraction (27%) consisting of proteins involved in DNA replication and repair (Table 1). Similar Cys motifs and thus potential Zn/Cys sites are present in more than 30 additional *D. deserti* proteins, including uncharacterized small proteins containing four or more cysteine residues (e.g. the radiation-induced 74-residue-long DdrS with two CXXC motifs) (Supplementary Table S1). In a *ddrS* mutant, no difference was observed for DdrO cleavage induction upon stress when compared to the wild-type strain (Supplementary Fig. S6). Taking into consideration all these data, we presume that zinc ions are released from various different proteins following disturbed redox homeostasis. Indeed, the list of identified Zn/Cys site-containing *D. deserti* proteins (Supplementary Table S1) contains homologs of at least 10 proteins known to release zinc in stress conditions: Hsp33, Trx2, FUR family proteins, DnaG, PriA, QueC, RpoC, ThrS, AlaS, ClpX^49,51^.

**Table 1:** DNA replication and repair proteins with zinc/cysteine sites in *D. deserti*.

Label	Gene	Description
Deide_00480	*priA*	Probable primosomal protein N' (ATP-dependent helicase PriA)
Deide_01610	*dnaX*	DNA polymerase III subunit gamma/tau
Deide_02040		DEAD/DEAH box helicase-like protein
Deide_04900	*dnaG*	DNA primase
Deide_06340	*recR*	Recombination protein RecR
Deide_06510		ATP-dependent DNA helicase
Deide_08980		Putative DNA/RNA helicase, SNF2 family
Deide_11320	*recQ*	ATP-dependent DNA helicase RecQ
Deide_12290	*ligA2*	DNA ligase 2
Deide_12660	*radA*	DNA repair protein RadA
Deide_12760	*uvrA1*	UvrABC system protein A (UvrA protein)
Deide_13810	*recO*	DNA repair protein RecO
Deide_16240	*fpg*	Formamidopyrimidine-DNA glycosylase (Fapy-DNA glycosylase)
Deide_21710		putative DNA polymerase III subunit delta'
Deide_1p00290	*ligA1*	DNA ligase 1
Deide_1p01280		ATP-dependent DNA helicase RecQ-like
Deide_2p02060	*uvrA2*	UvrABC system protein A (UvrA protein)

### The GAF-like domain of IrrE is not essential to induce DdrO cleavage

**Figure 5:**
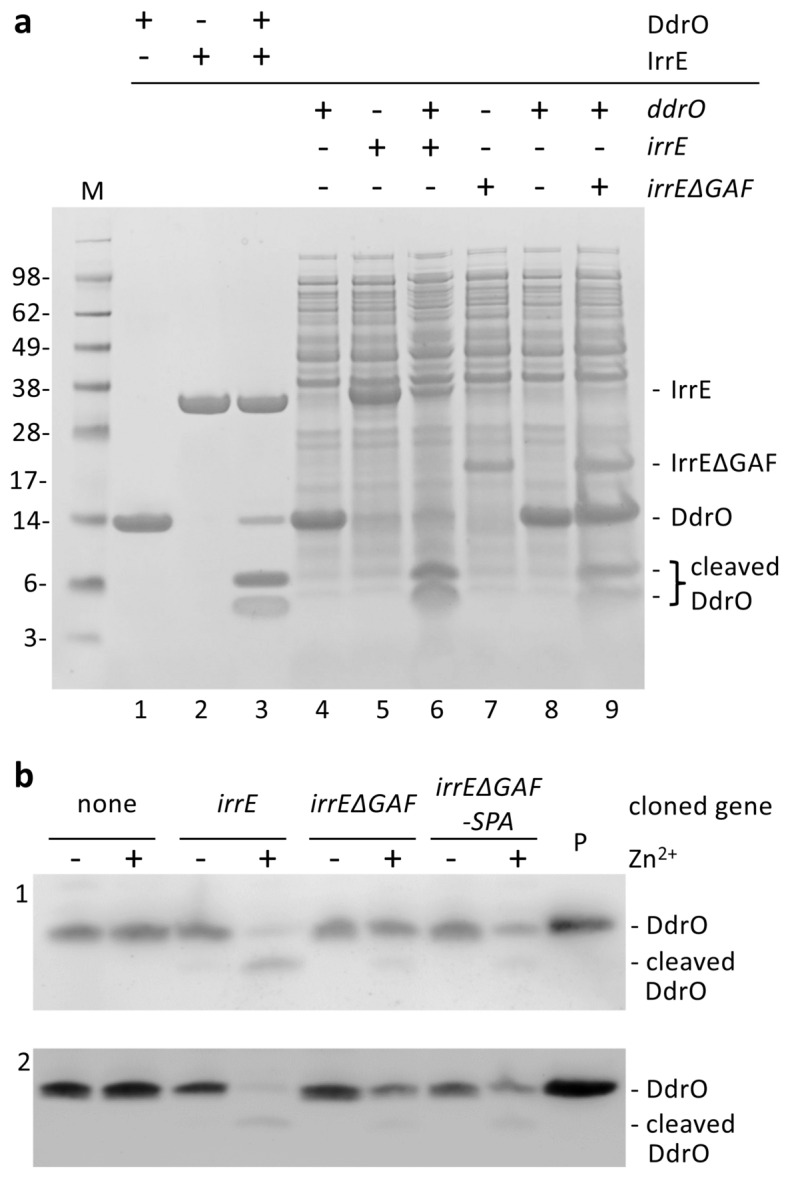
DdrO cleavage by a truncated IrrE lacking its GAF-like domain. (**a**) SDS-PAGE showing cleavage of DdrO when co-expressed with IrrE or IrrE lacking its GAF-like domain (IrrEΔGAF) in *E. coli* (lanes 4 to 9). The same gel contains purified DdrO (lane 1), IrrE (lane 2) and the products of in vitro cleavage (10 min 37 °C; lane 3). IrrE and IrrEΔGAF have an N-terminal His-tag, DdrO a C-terminal His-tag. M, molecular weight marker proteins (masses in kDa). (**b**) Western blots showing induced cleavage of DdrO by IrrEΔGAF in *D. deserti*. Strain RD42 (Δ*irrE*) was transformed with a derivative of plasmid pI3 containing the indicated cloned gene, and DdrO cleavage was analyzed after zinc shock (250 μM for 10 min). In IrrEΔGAF-SPA, the GAF-like domain is replaced by a SPA-tag. Except for the strain without cloned gene, induced DdrO cleavage is (faintly) visible in each strain (appearance of cleavage product and decrease of intact DdrO). The cropped blots (1 and 2) are from two independent series of experiments (uncropped blots are shown in Supplementary Fig. S9). A third independent experiment is shown in Supplementary Fig. S8. P, 20 ng purified DdrO.

The results described above indicate that IrrE protease activity is stimulated by increased availability of zinc ions following exposure to radiation and other agents that cause ROS production and redox imbalance. Then what could be the role of the C-terminal GAF-like domain of IrrE? GAF domains may bind small signaling molecules or may have a role in protein–protein interactions^25,26^. IrrE/DdrO-related systems are found in other bacteria, but a GAF-like domain is not present in all IrrE-like proteins^19^. Therefore, it is interesting to investigate whether or not this domain plays a role in relation with zinc signaling. When a truncated IrrE lacking this domain was co-expressed with DdrO in *E. coli*, DdrO cleavage was observed (Fig. 5a), showing that the GAF-like domain is not strictly necessary for the cleavage by the N-terminal zinc peptidase domain. Nevertheless, the cleavage was clearly less efficient than with entire IrrE. The truncated IrrE could not be purified from *E. coli*, indicating that the C-terminal domain has at least a structural role important for protein stability, and possibly contributes in the interaction with DdrO, as suggested by modeling of the IrrE-DdrO complex (Supplementary Fig. S7)^19^. IrrE lacking its C-terminal domain was also expressed, under control of the *irrE* promoter, in a *D. deserti irrE* deletion mutant. Strikingly, some DdrO cleavage was observed after exposure of the cells to zinc shock, showing that the GAF-like domain is not absolutely required for the induction of IrrE protease activity in *D. deserti* (Fig. 5b; Supplementary Fig. S8).

## Discussion

**Figure 6:**
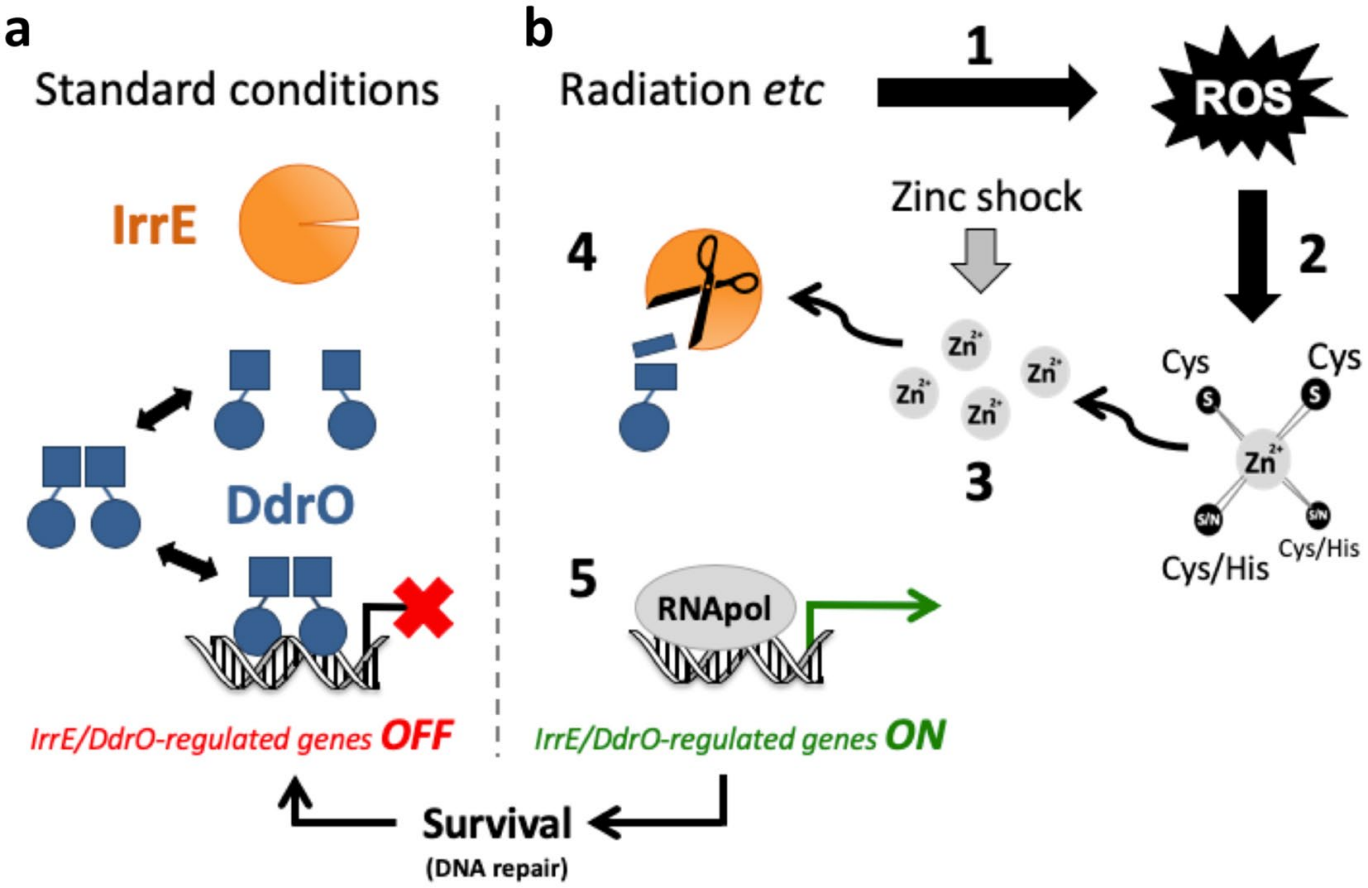
Proposed model of the IrrE/DdrO-controlled stress response mechanism. (**a**) Under standard conditions, repressor DdrO exist as a dynamic equilibrium between monomers and free and DNA-bound dimers. DdrO dimerization is mediated by its C-terminal domain. Binding of DdrO dimer to a conserved target DNA motif located in the promoter region of IrrE/DdrO-regulated genes represses their transcription. The majority of metalloprotease IrrE is inactive. (**b**) Exposure to conditions such as radiation generates oxidative stress through formation of ROS (step 1), which can cause oxidation of cysteine residues of zinc/cysteine sites in proteins (step 2), concomitantly causing release of zinc ions from these sites and a transient increase in the intracellular concentration of free, available zinc ions (step 3). The released zinc functions as second messenger, and increases the amount of active zinc-bound IrrE that cleaves the C-terminal domain of monomeric DdrO (step 4) abolishing its dimerization and shifting the DdrO equilibrium toward cleavable monomers. The diminished amount of DdrO leads to induced expression of the IrrE/DdrO-regulated genes (including several DNA repair genes and *ddrO* itself) (step 5). A zinc shock directly augments the intracellular free zinc ion concentration. Figure based on data obtained here and previously^14,17,19^.

The deinococcal *irrE* and *ddrO* genes have been described for the first time more than 15 years ago, with *irrE* being required for radiation resistance and radiation-induced expression of *recA*^53^, and *ddrO* as one of the genes highly induced after radiation or desiccation^16^. The precise function of IrrE and DdrO remained unknown for many years until we showed that IrrE and DdrO function together, which led to the description of a novel stress response mechanism where metalloprotease IrrE cleaves and inactivates transcriptional repressor DdrO when the cells are exposed to radiation^14^, thereby inducing expression of RDR regulon genes (e.g. *recA* and other DNA repair genes)^17^. Very recently we showed that DdrO consists of two domains: the predicted N-terminal DNA-binding domain, and a C-terminal dimerization domain showing a new fold^19^. Cleavage of this C-terminal domain by IrrE abolishes dimerization and DNA binding of DdrO. Insight in the protease activity of IrrE in *Deinococcus*, and its stimulation, was obtained in the present study, offering a more complete view of the regulatory mechanism (Fig. 6). We showed that DdrO cleavage by IrrE in *Deinococcus* cells is dependent on zinc ions, and, after having investigated several possible activation mechanisms, that this in vivo activity is stimulated directly by an increase in available zinc ions very likely resulting from modification in the cell redox status.

IrrE-mediated DdrO cleavage in *Deinococcus* is induced after exposure to radiation, desiccation, mitomycin C, H_2_O_2_ and zinc shock, indicating that these conditions share a common intracellular signal stimulating the cleavage. Radiation, desiccation, mitomycin C and H_2_O_2_ have in common that they induce DNA damage and oxidative stress^6^. The latter may involve formation of oxygen-radical species through the Fe^2+^-catalyzed Fenton reaction. However, the zinc shock-induced DdrO cleavage was not accompanied by DNA damage or ROS production and was not inhibited by an Fe^2+^ chelator.

Many proteins and enzymes bind Zn^2+^, this ion usually having a catalytic or structural role. At catalytic sites, such as in IrrE, the zinc ion is often bound to histidine, glutamic acid and aspartic acid residues. At structural sites, the Zn^2+^ is often bound to the thiolates of four cysteines (e.g. two pairs of the CXXC motif) or to a combination of cysteine and histidine residues^35,52^. Such zinc-binding residue pairs may also be part of a protein dimer interface^34^, as proposed, for example, regarding dimerization of the DNA repair protein SbcC through a zinc-bridge coordinated by CXXC motifs of two monomers^49^. Impaired redox homeostasis and increased ROS content can result in oxidation of the thiolates that coordinate Zn^2+^ and destroy the Zn^2+^-binding site, resulting in rapid release of zinc ions that can subsequently bind to other proteins^31,33,34^. Oxidation or other modifications of the zinc-bound thiolate may occur directly by various ROS or other reactive species^33,34,47,48^, or indirectly by upstream redox sensors that transmit oxidizing equivalents^54–58^. Hsp33, Trx2, FurS, RsrA, RslA and DksA are examples of bacterial proteins for which oxidative stress-induced zinc release has been reported^49,56^. These proteins have been studied because of their role in sensing and responding to oxidative stress. Their Zn/Cys centers function as redox switches, and oxidation/reduction regulates the activity of these proteins. For example, under non-stress condition the four conserved Cys residues of Hsp33 bind a zinc ion and Hsp33 is inactive. Oxidants such as H_2_O_2_ and hydroxyl radicals oxidize the Zn/Cys center, inducing zinc release and intramolecular disulfide bond formation, and Hsp33 can acquire its chaperone activity after conformational rearrangement^31,59^. Besides the redox-induced modification of the activity of these proteins, the released zinc may participate in other pathways. Of note, Hsp33 proteins are highly conserved in *Deinococcus*^7^. Although there are lower levels of Fe^2+^-catalyzed oxidative protein damage in *Deinococcus* compared to radiation-sensitive species^9^, it can be assumed that proteins like Hsp33 function in the same way in *Deinococcus* as in other bacteria, that is, involving oxidation of Cys residues (with concomitant release of zinc ions). Many other *Deinococcus* proteins, such as FUR family members and Trx2 (see Results), may release zinc upon oxidation of their Zn/Cys sites.

Zinc shock and oxidative stress have thus in common that they transiently augment the intracellular concentration of free zinc ions (i.e. not bound to proteins). We propose that released zinc acts as a second messenger that signals redox imbalance and, by becoming more available to IrrE, increases the amount of active zinc-bound IrrE to induce the radiation response (Fig. 6). Under standard conditions, bacterial cells maintain an extremely low concentration of free zinc (less than one atom per cell), and most zinc ions are bound to proteins or other molecules^60^. Even a small increase in free zinc can thus be a very potent signal, which can be sensed by zinc-responsive proteins (e.g. IrrE in *Deinococcus*). A role of released zinc as second messenger in regulation of enzyme activities has been described for eukaryotic cells^33–35^, but such a role in induction of an oxidative stress/DNA damage response in bacteria has, to our knowledge, not been described previously. Remarkably, many DNA replication and repair proteins have Zn/Cys sites (Table 1). It is tempting to speculate that destruction of their zinc-binding sites inhibits their function, while the mobilized zinc stimulates IrrE activity and expression of DNA repair genes.

Genes encoding IrrE/DdrO-like protein pairs consisting of a putative metalloprotease (COG2856; ImmA/IrrE family) and a transcriptional regulator (XRE family) have been identified in many bacteria and bacteriophages^14,27,29^. Only a few have been studied experimentally, which showed a stress-induced inactivation of the XRE family repressor by the COG2856 domain-containing protein, resulting in induction of prophage or transposon excision^27,28,61,62^. In each case, however, it is unclear how stress activates the COG2856 protein. Of note, these other studied COG2856 domain proteins are smaller than IrrE and, unlike IrrE, do not contain an additional GAF-like domain^19^. As we observed protease activation of truncated IrrE lacking the GAF-like domain, this supports the proposed mechanism of IrrE activation and suggests that oxidative stress-induced release of zinc may also be involved in activation of GAF-lacking IrrE-like proteins. One of these metalloproteases, MpaR in *Listeria monocytogenes*, was found to be activated during intracellular growth of this pathogen in macrophages^61^, during which bacteria experience oxidative and nitrosative stress. Activated MpaR triggers excision of a prophage, thereby promoting virulence of *L. monocytogenes*^61^. The potential role of zinc signaling in MpaR activation is supported by another recent study that showed that nitric oxide produced by macrophages causes release of zinc from Zn/Cys sites of at least 15 proteins in the pathogen *Salmonella enteritica*^51^, including homologs of eight Zn/Cys site-containing proteins conserved in *Deinococcus*. The COG2856 protein Rir in *Streptococcus thermophilus*, a species used dairy industry, is involved in prophage induction causing cell lysis by interacting with and preventing oligomerization of repressor Crh without proteolytic cleavage of the latter, despite the presence of a predicted HEXXH zinc-binding active site in Rir^28^. In this case, it can be hypothesized that zinc binding stabilizes Rir or the Rir/Crh interaction. Taken together, a role for zinc as second messenger following stress exposure of bacteria may be more universal than currently recognized.

## Methods

### Growth media and culture conditions

The strains used in this study are listed in Supplementary Table S2. *E. coli* was grown in Luria–Bertani (LB) at 37 °C. *D. deserti* and *D. radiodurans* were grown at 30 °C in tenfold diluted Tryptic Soy Broth (Fluka, Sigma-Aldrich, T8907) (TSB/10) supplemented with trace elements^22^ or on agar (1.6%) plates containing the same growth medium. Antibiotics were used at the following concentrations for *D. deserti*: streptomycin, 10 µg ml^−1^; kanamycin, 10 µg ml^−1^; chloramphenicol, 2 µg ml^−1^. For *E. coli*, the antibiotics concentrations were: kanamycin, 50 µg ml^−1^; ampicillin, 100 µg ml^−1^.

### Plasmids and DNA manipulations

Plasmids used are listed in Supplementary Table S3. Standard molecular biology techniques were used to construct plasmids. PCR products obtained with primers that included restriction sites were cloned in pCR4Blunt-TOPO prior to recloning in the desired vector. All cloned PCR fragments and site-directed mutations were analyzed by DNA sequencing to verify absence of potential PCR errors. Primer sequences are listed in Supplementary Table S4. *D. deserti* genes cloned in the *E. coli* expression vectors pET-TEV and pET22b are under control of the T7 promoter. Point mutations in IrrE (M18V, M243L and C116A) were introduced in pRD48, which contains an XbaI-HindIII fragment with *irrE* and its promoter region, using the QuikChange II Site-Directed Mutagenesis Kit (Stratagene) and appropriate primers. Then the XbaI-HindIII fragments with the mutations were recloned into pI3. The cloned XbaI-HindIII fragment in pI3-irrEΔGAF contains the promoter of *irrE* and the region encoding IrrE lacking its C-terminal 114 residues corresponding to the GAF-like domain. A similar DNA fragment is present in pI3-irrEΔGAF-SPA, except that it encodes an IrrE protein with its GAF-like domain replaced by a SPA-tag (69 aa) to render the truncated IrrE potentially more stable. The fusion of IrrEΔGAF with the SPA-tag was obtained with fusion PCR. The different pI3 plasmids were used to transform *D. deserti* RD42 as described previously^46^.

### Exposure to UV, H_2_O_2_, MMC or zinc shock

*Deinococcus* strains grown to OD_600_ 0.4 were exposed to zinc shock, H_2_O_2_, MMC or UV radiation (gamma radiation facilities in our institute have been dismantled). For UV irradiation, portions of 5 ml of the culture were exposed (in a Petri dish without lid) to UV-C (254 nm) using a Bio-Link BLX (Vilber Lourmat). After UV irradiation, cells were incubated at 30 °C for 10 min (or other times if indicated in the figures). For other exposures, H_2_O_2_ (Riedel-de Haën, 18312), MMC (Sigma-Aldrich, M0440), ZnCl_2_ (Sigma-Aldrich, 229997) or MnCl_2_ (Prolabo, 25222.233) was added to 10 ml of culture and incubation was continued (final concentrations and incubation times as indicated in the figures).

Exposure to these agents was also performed in the presence of metal chelators (TPEN for Zn^2+^, or DIP for Fe^2+^) or antioxidants (thiourea or TEMPOL) that were added at final concentrations as used previously^41,63,64^. For this, bacteria were grown to OD_600_ 0.4 and then either 10–50 µM TPEN (N,N,N′,N′-tetrakis(2-pyridinylmethyl)-1,2-ethanediamine; Sigma-Aldrich, P4413), 500 µM DIP (2,2′-dipyridyl; Sigma-Aldrich, D216305), 150 mM thiourea (Sigma-Aldrich, T7875), or 10 mM TEMPOL (4-hydroxy-2,2,6,6-tetramethylpiperidin-1-oxyl; Sigma-Aldrich, 581500) was added and incubation was continued for 30 min before the cells were exposed to zinc shock, UV, H_2_O_2_ or MMC.

After the incubations, cells were collected by centrifugation for 1 min at 10,000*g* and directly lysed by heating for 10 min at 95 °C in Novex NuPAGE LDS Sample Buffer supplemented with Novex NuPAGE Sample Reducing Agent (Invitrogen) (volume corresponding to 100 µl sample buffer for 1 ml of culture with an OD_600_ of 1) and by passing through a syringe needle, and then frozen at − 20 °C. Protein separation by SDS-PAGE (20 μl sample per lane) and immunoblotting to analyze DdrO cleavage were performed as described previously^14^. A lane with 20 ng DdrO, purified as described^17^, was included on the Western blots. The experiments were performed in triplicate (biological replicates), and representative results are shown.

### Co-expression of IrrE or IrrEΔGAF with DdrO in *E. coli*

Two plasmids were used for co-expression in *E. coli*: a pET-TEV derivative encoding IrrE or IrrEΔGAF (both with N-terminal His-tag) and a pET22b derivative encoding DdrO (with C-terminal His-tag). *E. coli* BL21 (AI) cells freshly transformed with the two plasmids were grown at 37 °C overnight to saturation in 10 ml of LB medium containing kanamycin and ampicillin. This pre-culture was used to inoculate (start OD_600_ 0.05) 100 ml of LB medium with the antibiotics and grown at 37 °C with aeration. At OD_600_ of 0.6–0.7, IPTG (0.1 mM final concentration) and L-arabinose (0.2% final concentration) were added and the cells were further incubated at 37 °C for 3 h. Five hundred µl of induced cells were taken, centrifuged (10,000*g*, 2 min, 4 °C) and re-suspended in Novex NuPAGE LDS Sample Buffer (100 µl of sample buffer for 1 ml of culture at OD_600_ 1), and heated for 10 min at 95 °C. Samples (20 μl per lane) were loaded on SDS-PAGE gels (Novex NuPAGE 10% Bis–Tris Gels; Invitrogen), and migrated in 1X NuPAGE MES SDS Running Buffer (Invitrogen) for 1 h at 130 V. In vitro cleavage control was performed as described previously^14^. The proteins were visualized by staining with Imperial protein stain (Pierce).

### Induced mutagenesis

*D. deserti* strains were grown to OD_600_ 0.4 and then 250 µM ZnCl_2_ or 10 mM H_2_O_2_ or nothing (control) was added, and cultures were incubated further for 10 min at 30 °C. One part (1 ml) of the cultures was taken to prepare samples for immunoblotting to analyze DdrO cleavage as described above. The other part of the cultures was centrifuged for 5 min at 5000 g, and the cell pellets were re-suspended in fresh growth medium and incubated for 20 h at 30 °C with shaking to permit fixation of mutations. Then 1 ml of each culture were spread in duplicate on plates with 10 µg ml^−1^ rifampicin. In parallel, 0.1 ml of serial dilutions 10^−6^ and 10^−7^ were spread in duplicate on plates without rifampicin. The entire experiment was performed in triplicate (biological replicates). Colony-forming units (CFU) were determined after incubation for 3–4 days at 30 °C. The mutation frequency was calculated by the ratio "number of Rif^R^ CFU/ml" / "total number of CFU/ml".

### Zinc measurements with fluorescent probe FluoZin-3

Experiments to detect free intracellular Zn^2+^ were based on a previously described protocol^65^. *D. deserti* was grown to OD_600_ 0.4. Bacteria were resuspended in fresh TSB/10 and then incubated with 5 µM FluoZin‐3, AM, cell permeant (Thermo Fisher Scientific, F24195) for 60 min at room temperature in the dark to allow complete de‐esterification of intracellular acetoxymethyl (AM) esters. The bacteria were then washed three times in TSB/10. Fluorescence measurements were performed in a microplate at 28 °C and using a spectrofluorimeter (Infinite 200 Pro; Tecan). After excitation of the sample at 485 nm, fluorescence emission was recorded at 519 nm before and after addition of H_2_O_2_ or ZnCl_2_.

### ROS measurements with fluorescent probe H_2_DCFDA

ROS detection using H_2_DCFDA (2′,7′-Dichlorodihydrofluorescein diacetate; Sigma-Aldrich, D6883) was performed after adaptation of a previously described method^66^. *D. deserti* was grown to OD_600_ 0.4. Bacteria were washed once in 0.85% KCl and then incubated with 25 µM H_2_DCFDA for 60 min at room temperature in the dark, and then washed again in 0.85% KCl. Fluorescence measurements were performed in a microplate at 28 °C and using a spectrofluorimeter (Infinite 200 Pro; Tecan). After excitation of the sample at 480 nm, fluorescence emission was recorded at 526 nm before and after addition of H_2_O_2_ or ZnCl_2_.

### Construction of *D. deserti* gene deletion mutants

*D. deserti* strains in which *ddrS* (Deide_04721), *bshA* (Deide_11680) or *bshC* (Deide_13730) are deleted and replaced by a kanamycin resistance gene, were constructed as described previously for other deletion mutants^46^. Briefly, DNA fragments corresponding to upstream and downstream regions of the gene to be deleted, were cloned upstream and downstream of a kanamycin resistance cassette in a pUC19 derivative. Resulting plasmids, which do not replicate in *D. deserti*, were introduced in *D. deserti*, and double homologous recombination events and complete deletion of the gene of interest were verified by diagnostic PCR.

## Supplementary Information

Supplementary Information.

## Data Availability

The IrrE and DdrO genes and proteins in this study correspond to SwissProt entries C1CZ84 and C1CYP4, respectively. All data that support the findings of this study are available from the corresponding author on request.
