# Supplementary material for: Redox signaling through zinc activates the radiation response in Deinococcus bacteria
Source: Sci Rep. 2021 Feb 25;11:4528. doi: 10.1038/s41598-021-84026-x (PMC7907104; doi:10.1038/s41598-021-84026-x)
Supplement: Supplementary file 1 — Supplementary Information. [file 41598_2021_84026_MOESM1_ESM.pdf]

Supplementary Information for:

Redox Signaling through Zinc Activates the Radiation Response in *Deinococcus* Bacteria

Romaric Magerand,[1] Pascal Rey,[2] Laurence Blanchard,[1] Arjan de Groot[1,*]

[1]Aix Marseille Univ, CEA, CNRS, BIAM, Molecular and Environmental Microbiology Team, Saint Paul-Lez-Durance, F-13108, France

[2] Aix Marseille Univ, CEA, CNRS, BIAM, Plant Protective Proteins Team, Saint Paul-Lez-Durance, F-13108, France

* Corresponding author: Arjan de Groot; **Email:** nicolaas.degroot@cea.fr

This file includes:

Figures S1 to S9

Tables S1 to S4

Supplemental References

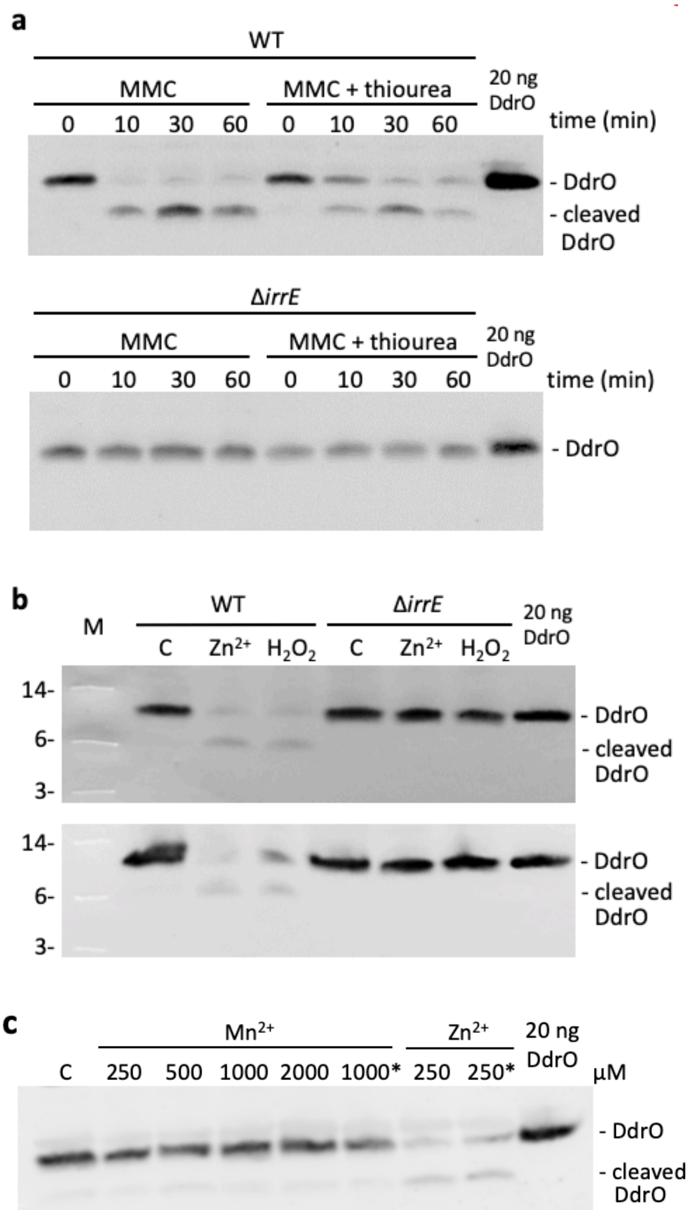

Fig. S1. MMC-, H_2O_2- and Zn^{2+}-induced DdrO cleavage is IrrE-dependent. **(a)** Western blots analyzing DdrO cleavage in *D. deserti* wild type (WT) or *irrE* deletion mutant after exposure to MMC (1 µg/ml). Like the experiment shown in main text Figure 1, this independent experiment also shows less efficient MMC-induced cleavage in the presence of the antioxidant thiourea. **(b)** Cleavage after exposure to H_2O_2 (10 mM for 10 min) or $ZnCl_2$ (250 µM for 10 min). C, untreated control. Results of two independent series of experiments are shown. IrrE-dependent DdrO cleavage after exposure to zinc shock or radiation was shown previously [1,2]. **(c)** Mn^{2+} shock does not induce DdrO cleavage. Cells were exposed to the indicated concentrations of Mn^{2+} or Zn^{2+} for 10 min. Also TPEN (50 µM) was added to the cells analyzed in the two lanes marked with an asterisk (*). Each cropped blot corresponds to a single independent blot; the five uncropped blots are shown in Fig. S9.

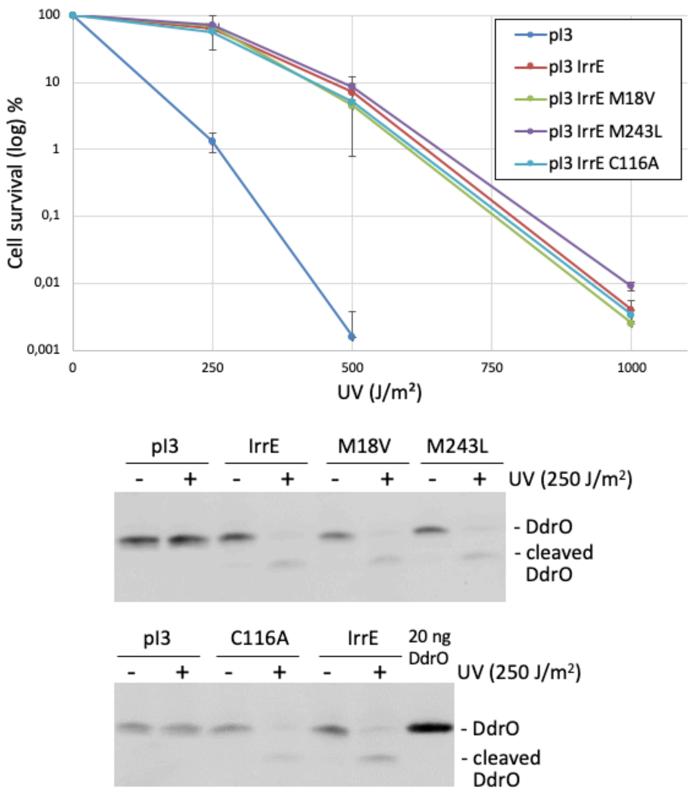

Fig. S2. The highly conserved methionine and cysteine residues in IrrE are not required for its activation**.** A multiple alignment (not shown) of 50 IrrE proteins of different *Deinococcus* species revealed that two methionine residues (M18 and M243) and one cysteine (C116) of *D. deserti* IrrE are strictly conserved in each deinococcal IrrE (except for *D. maricopensis* IrrE that has a leucine at the position corresponding to M243). In the IrrE structure, M18 and C116 are close to the zinc ion binding active site, and M243 is a surface residue in the GAF-like domain. These residues were mutated as indicated. The wild type or mutated *irrE* genes were cloned in plasmid pI3, and the pI3 derivatives were introduced in *D. deserti* strain RD42 that lacks its chromosomal *irrE*. To determine survival after UV irradiation, 20 µl volumes of serial dilutions of cultures (OD_{600} 0.4) were spotted on plates, which, after drying, were exposed to UV. Survival data are the average of three independent experiments (biological replicates) and the error bars are standard deviation. The survival curves and Western blots show that these sulfur-containing residues (and their potential oxidation) are not required for radiation resistance and induction of DdrO cleavage. Noteworthy, the residue corresponding to M18 is erroneously annotated as the N-terminal residue of many IrrE proteins in sequence data banks. The results with the M18V mutation (ATG mutated into the non-start codon GTC) once more show that M18 does not correspond to the N-terminus/translation initiation of IrrE (see also Ludanyi *et al*, 2014[1]). Each cropped blot corresponds to a single independent blot; the two uncropped blots are shown in Fig. S9.

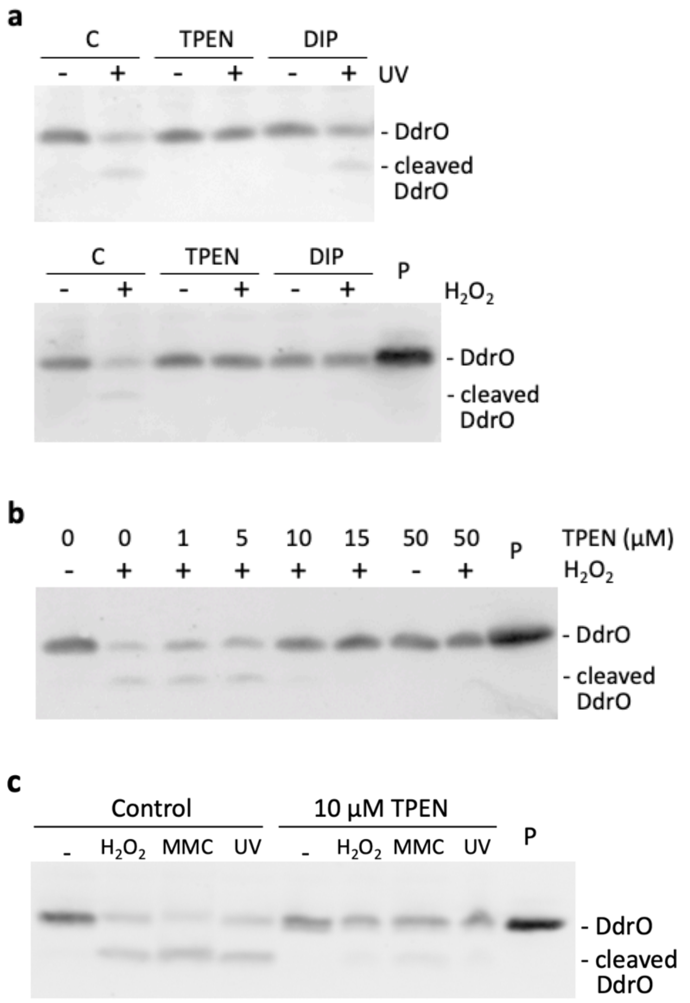

Fig. S3. IrrE-mediated DdrO cleavage in *D. radiodurans* and *D. deserti* is dependent on zinc ions. **(a)** Western blots showing DdrO cleavage in *D. radiodurans* after exposure (+) or not (-) to UV (500 J/m^2) or H_2O_2 (10 mM for 10 min) in the presence of Zn^{2+} chelator TPEN (50 µM) or Fe^{2+} chelator DIP (500 µM). As in *D. deserti* (main text Figure 2), DIP inhibits DdrO cleavage after exposure to H_2O_2 only, whereas TPEN inhibits the cleavage after each applied stress. C, controls without metal chelator. P, 20 ng purified DdrO. **(b)** DdrO cleavage in *D. deserti* after exposure to H_2O_2 (10 mM for 15 min) in the presence of different TPEN concentrations. **(c)** DdrO cleavage in *D. deserti* after exposure to H_2O_2 (10 mM for 10 min), MMC (1 µg/µl for 10 min) or to UV (250 J/m^2) in the presence or not of 10 µM TPEN. Each cropped blot corresponds to a single independent blot; the four uncropped blots are shown in Fig. S9.

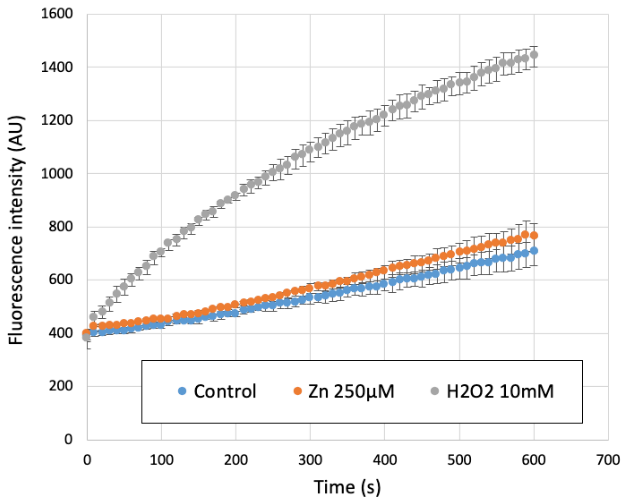

Fig. S4. The zinc shock does not induce ROS production in *D. deserti*. ROS was detected with fluorescence spectroscopy using H_2DCFDA, a cell permeant reagent that, after deacetylation by cellular esterases, reacts with ROS into a fluorescent compound. At time point 0, either $ZnCl_2$ (250 µM), H_2O_2 (10 mM) or nothing (control) was added to wild-type *D. deserti* cells, and fluorescence was recorded. The results are the average of three independent experiments (biological replicates) and the error bars are standard deviation.

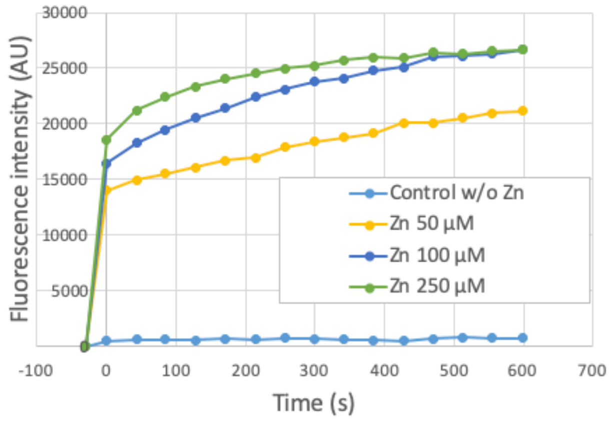

Fig. S5. Detection of free intracellular zinc in *D. deserti* after zinc shock. Upon addition of $ZnCl_2$ to the cells, increase of free intracellular zinc was detected using FluoZin-3 AM cell permeant after its de-esterification in the cells. The values are corrected for the fluorescence observed after the incubation for 60 min with FluoZin-3 AM but before treatment with $ZnCl_2$. w/o, without.

Fig. S6. DdrO cleavage and re-accumulation is similar in wild-type, *ddrS* and bacillithiol biosynthesis mutant strains. Each cropped blot corresponds to a single independent blot; the 19 uncropped blots are shown in Fig. S9. *This page and next four pages for Figs. S6a to S6e.*

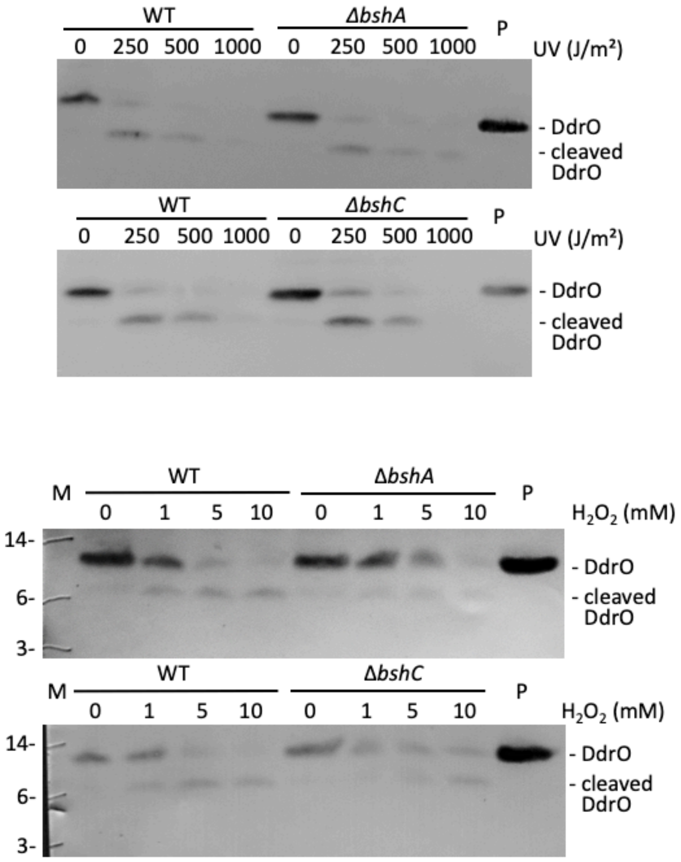

Fig. S6a. Western blots showing DdrO cleavage in *D. deserti* wild-type, Δ*bshA* and Δ*bshC* strains after exposure to H_2O_2 (10 min) or UV. P, 20 ng purified DdrO.

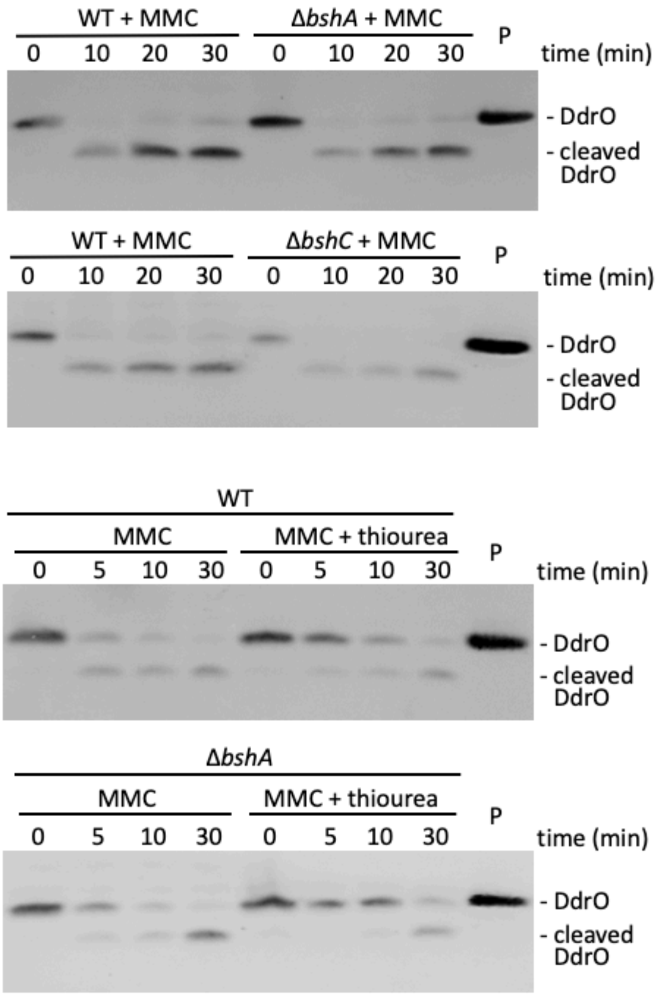

Fig. S6b. DdrO cleavage in *D. deserti* wild-type, Δ*bshA* and Δ*bshC* strains after exposure to 1 µg/ml MMC (top 2 blots). As in the wild type, cleavage induced by MMC (0.5 µg/ml) in the *bshA* mutant is less efficient in the presence of the antioxidant thiourea (150 mM) (bottom 2 blots; the "thiourea-blot" for the wild type is the same as shown in main text Figure 1). P, 20 ng purified DdrO.

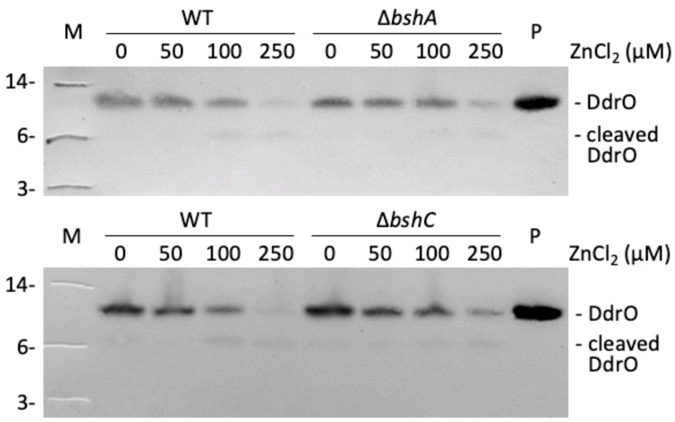

Fig. S6c. DdrO cleavage in *D. deserti* wild-type, Δ*bshA* and Δ*bshC* strains after zinc shock (10 min). P, 20 ng purified DdrO.

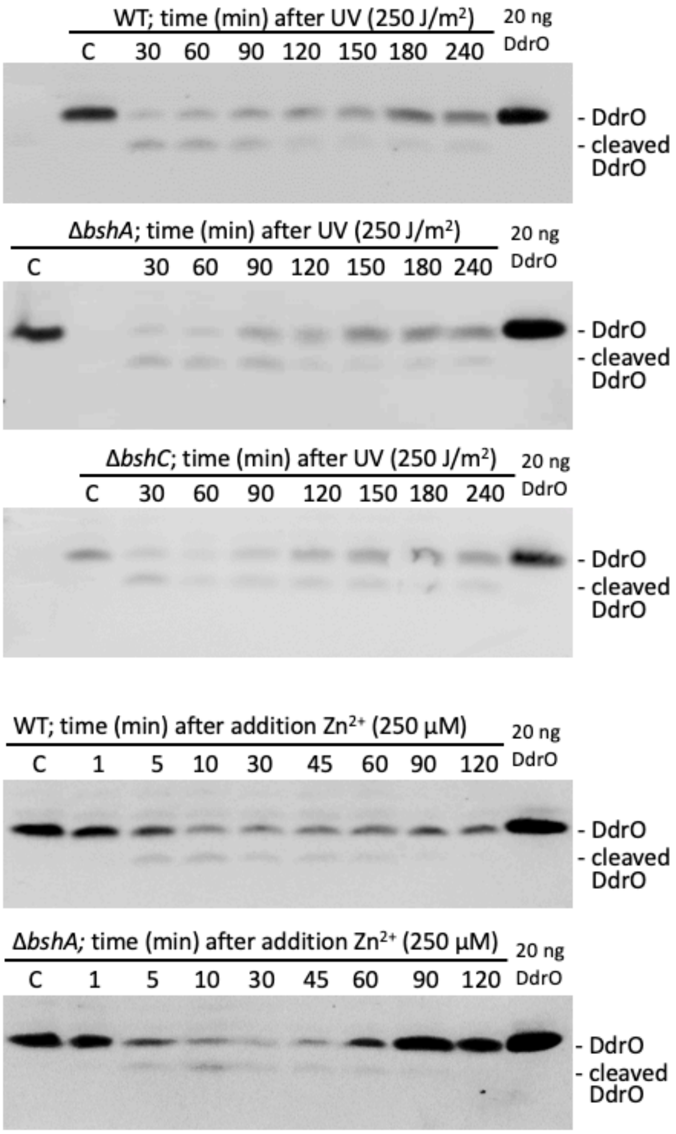

Fig. S6d. DdrO cleavage and re-accumulation in *D. deserti* wild-type, Δ*bshA and* Δ*bshC* strains after exposure to UV and zinc shock. C, untreated control cells.

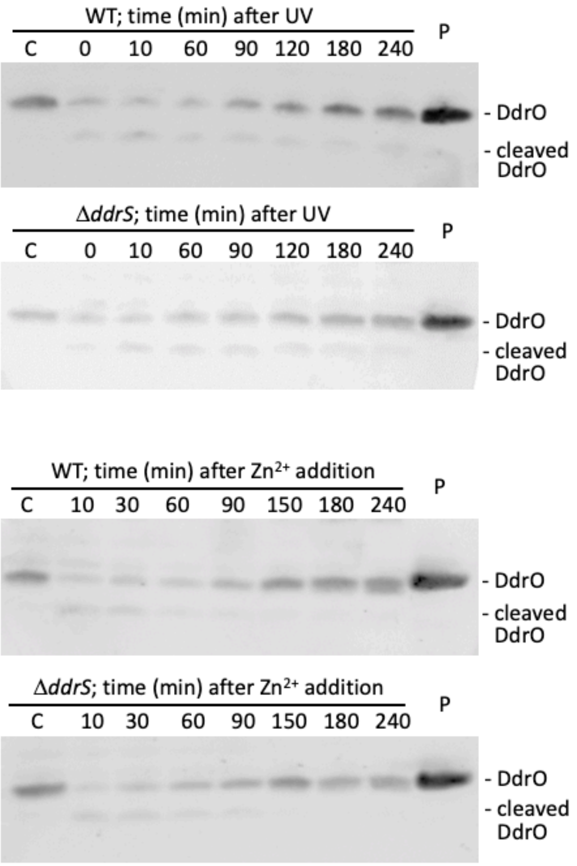

Fig. S6e. Western blots showing cleavage and re-accumulation of DdrO in *D. deserti* wild-type and Δ*ddrS* strains after exposure to UV (250 J/m^2) or zinc shock (250 µM). C, untreated control cells. P, 20 ng purified DdrO.

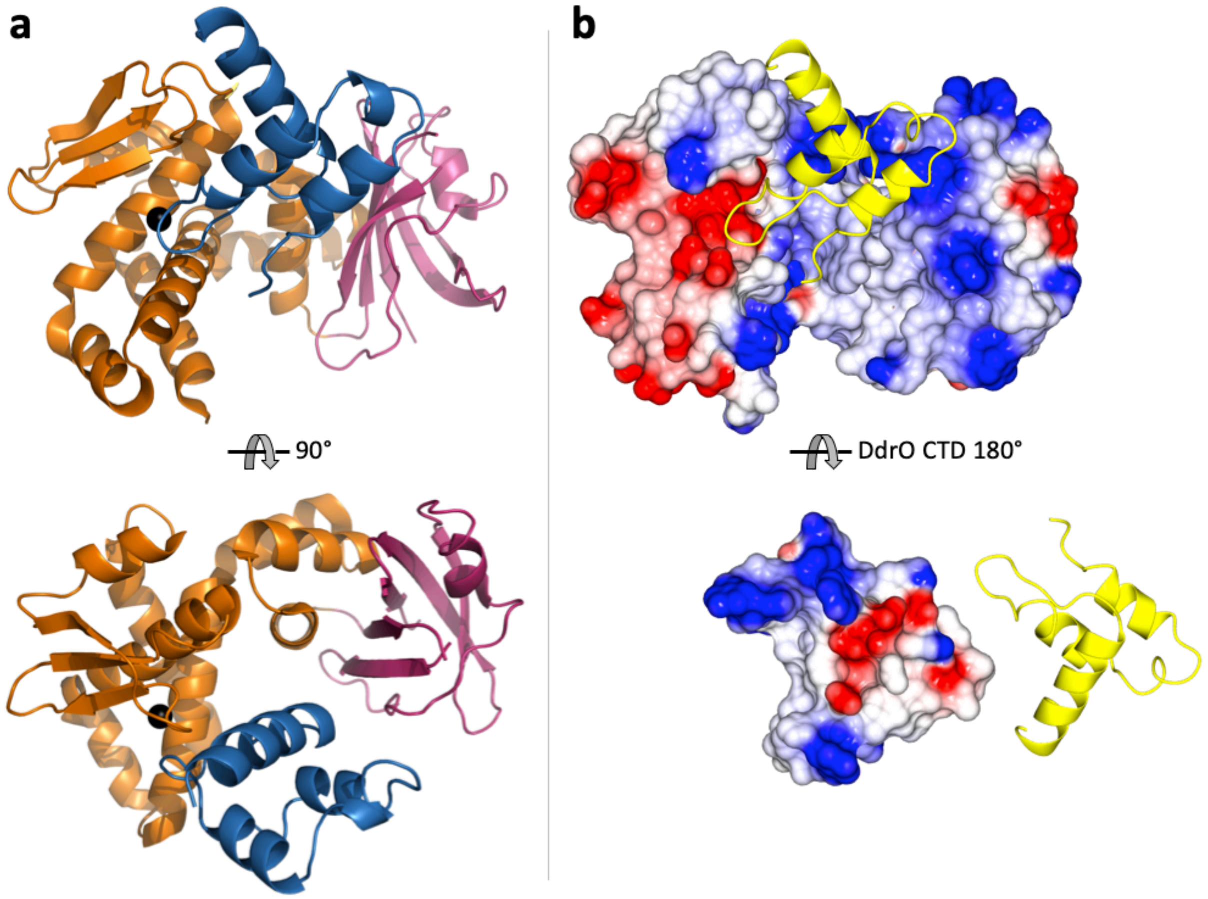

Fig. S7. Structural model suggesting contribution of the GAF-like domain of IrrE in interaction with DdrO. Molecular docking calculations were performed previously, and the model shown was found with two different docking tools[3]. **(a)** Two orientations of the complex of IrrE and the C-terminal domain (CTD) of DdrO. The COG2856 zinc peptidase domain, the zinc ion, and the C-terminal GAF-like domain are shown in orange, black and pink, respectively. DdrO CTD is shown in blue. **(b)** Surfaces of IrrE (top) and DdrO CTD (bottom, after rotation) coloured by electrostatic surface potential. For clarity, DdrO CTD is shown (top) or also shown (bottom) as yellow ribbon. Three-dimensional structure images were generated using PyMOL (PyMOL Molecular Graphics System, Version 2.0 Schrödinger, LLC) **(a)** and CCP4mg software[4] **(b)**.

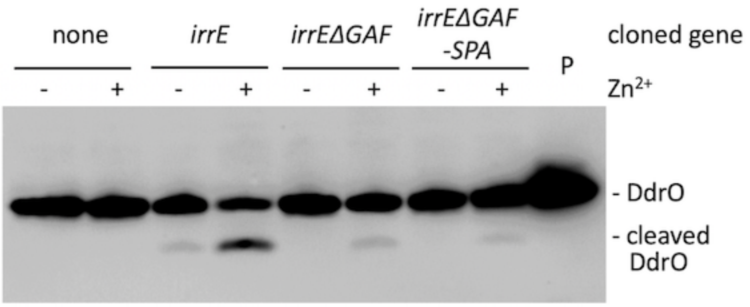

Fig. S8. Additional Western blot showing induced cleavage of DdrO by IrrE lacking its GAF-like domain in *D. deserti*. The blot was overexposed to better visualize cleaved DdrO. P, 20 ng purified DdrO. The cropped blot corresponds to a single independent blot; the uncropped blot is shown in Fig. S9.

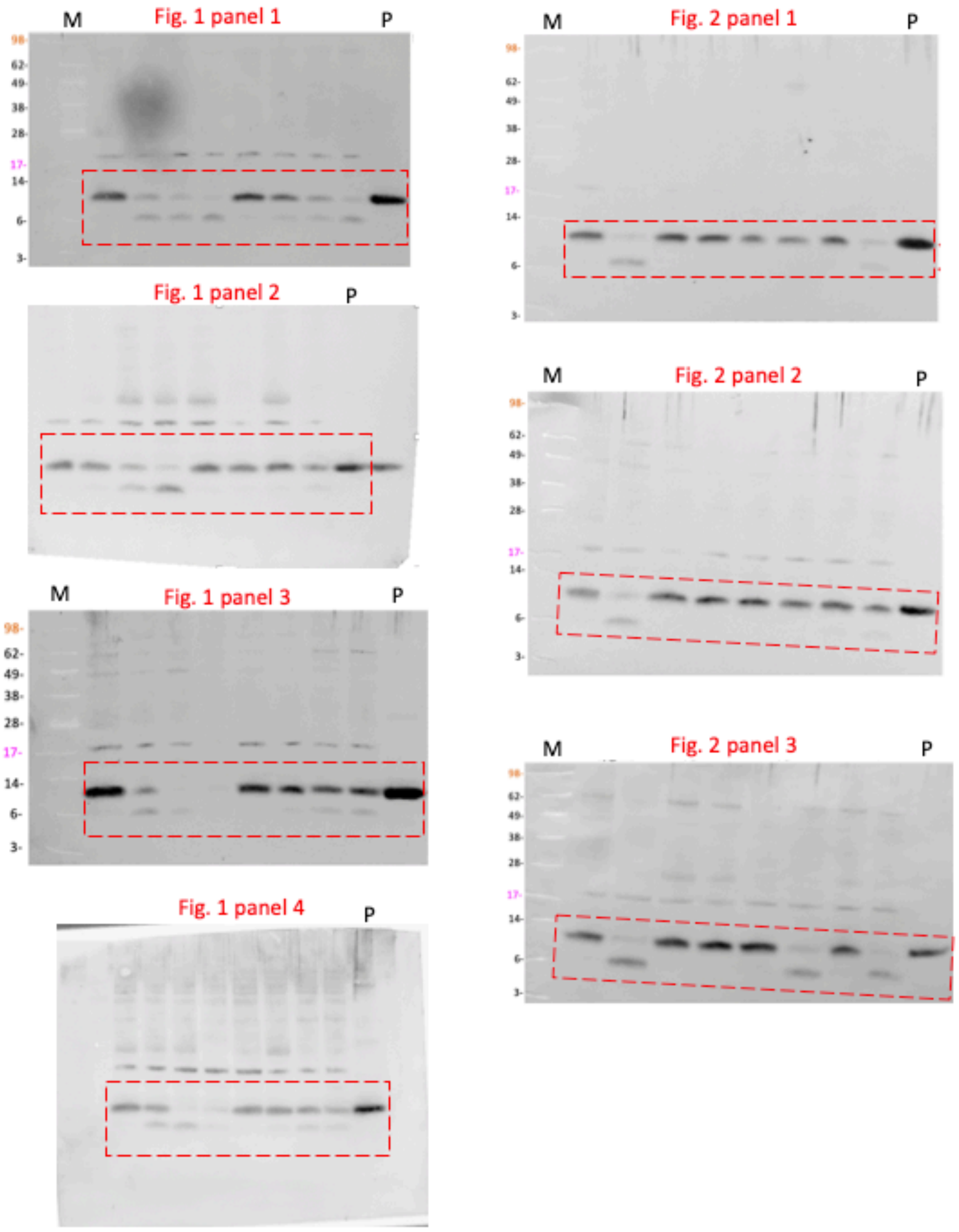

Fig. S9. *(and next 5 pages)* Uncropped Western blots. The 13 uncropped blots of the main text figures are shown first, followed by the 31 uncropped blots of the supplementary figures. Red boxes correspond to the cropped portions of the blots in the indicated figures. To improve the detection of the small low abundance protein DdrO (and of cleaved DdrO) compared to non-specific signals, for a few supplementary blots only the lower half of the membrane was incubated with the antiserum (i.e., Figs. S1c, S3a, S3c, S8). M, molecular weight marker proteins (masses in kDa). P, 20 ng purified DdrO.

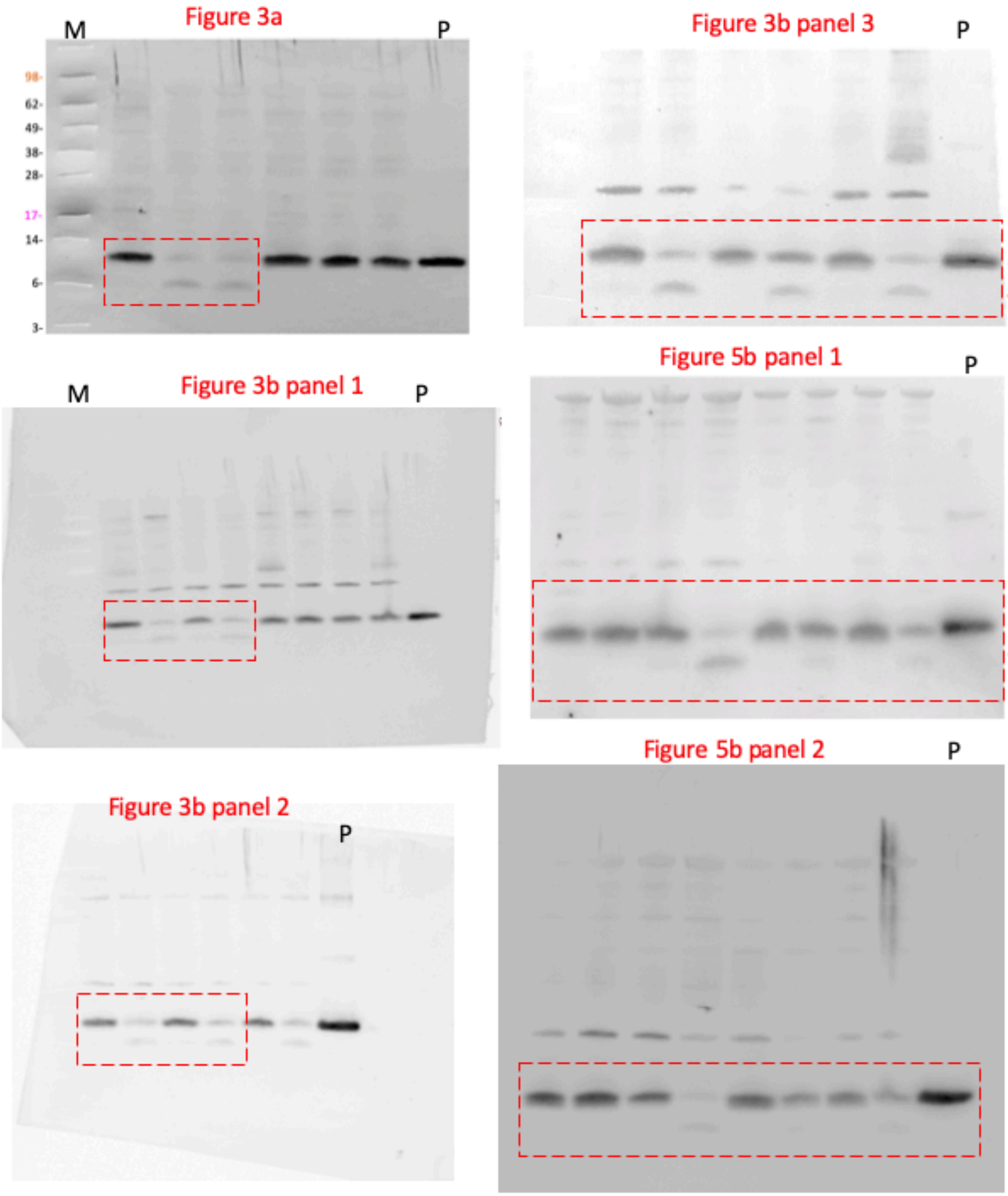

Fig. S9. *(continued)*

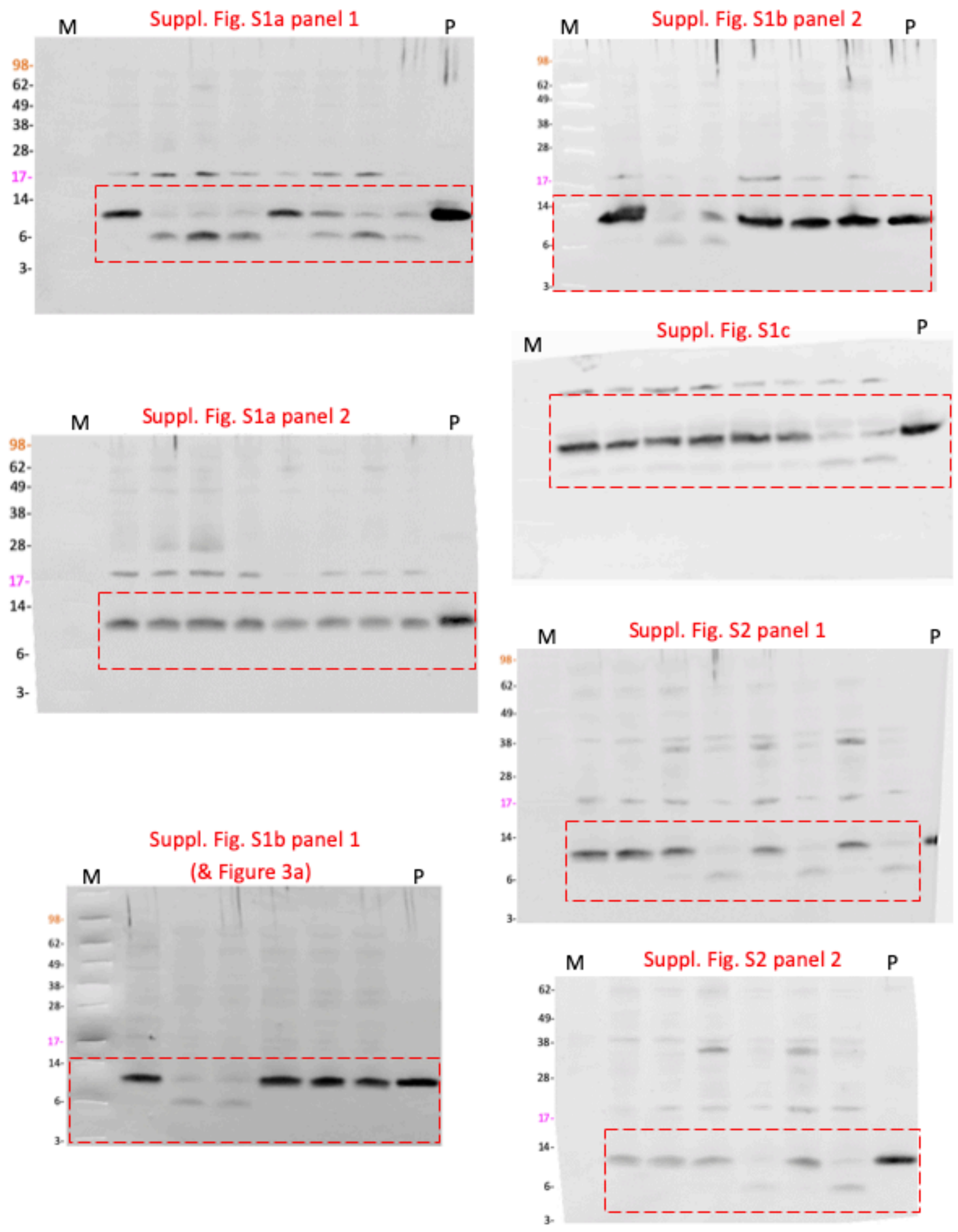

Fig. S9. *(continued)*

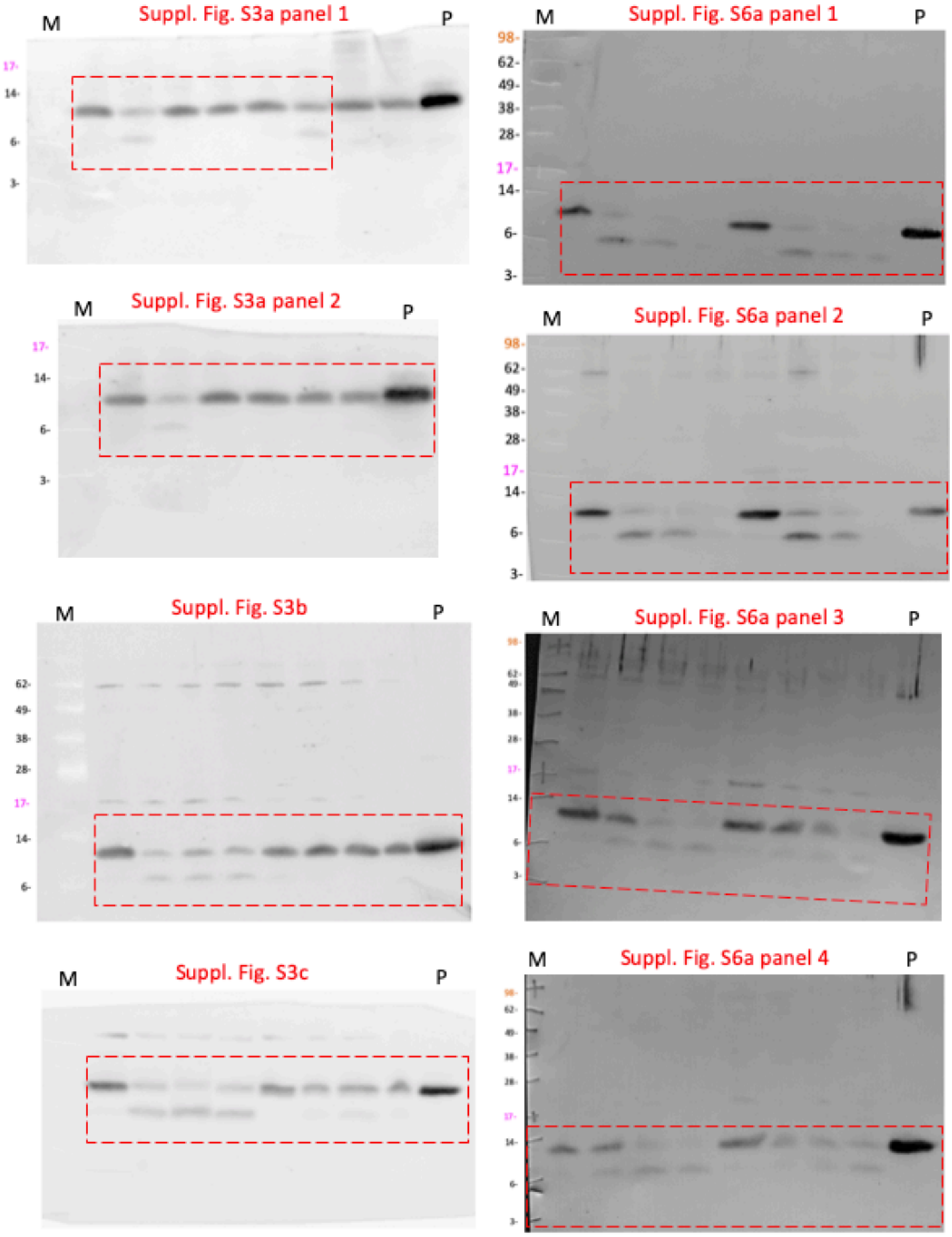

Fig. S9. *(continued)*

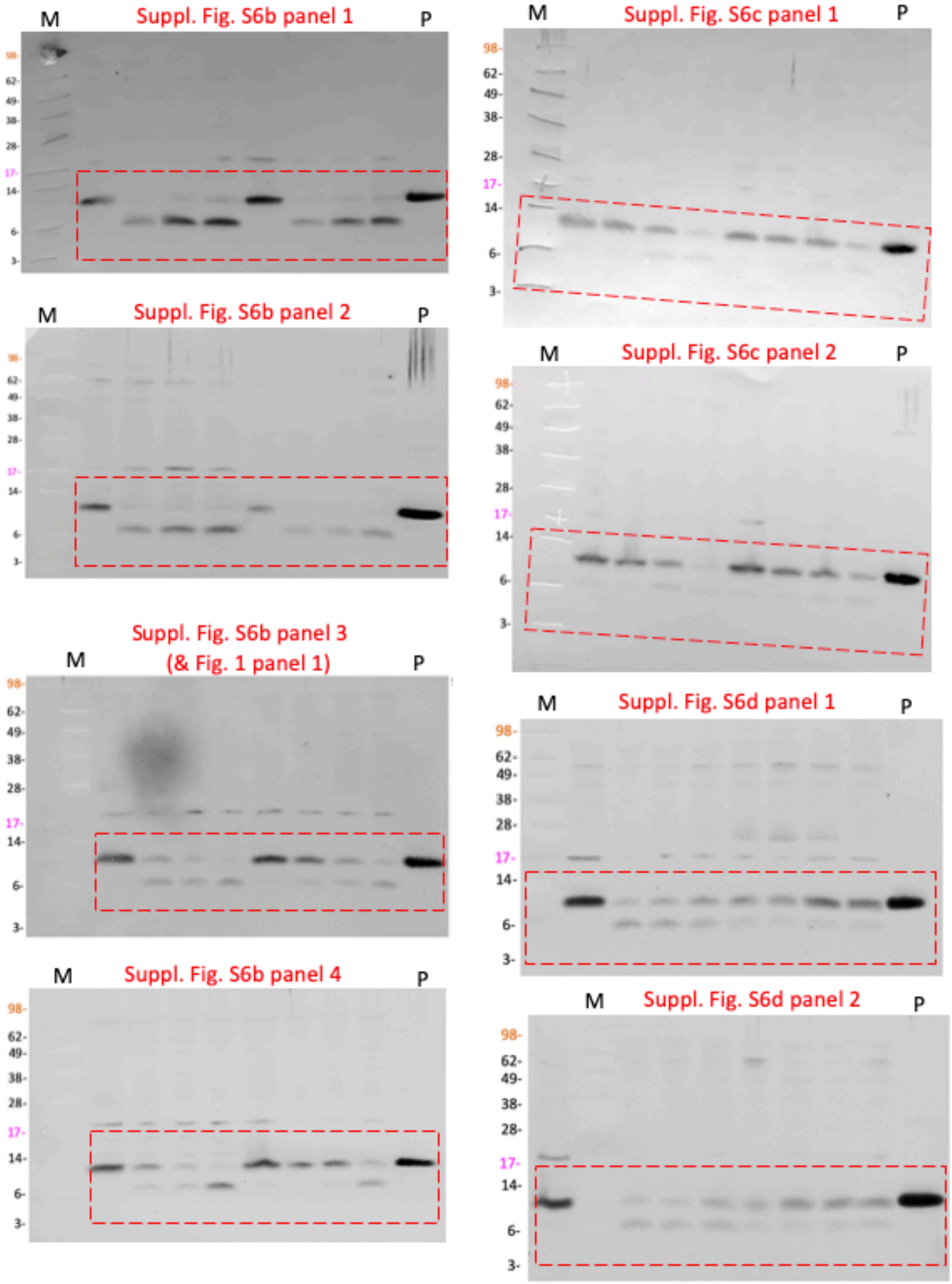

Fig. S9. *(continued)*

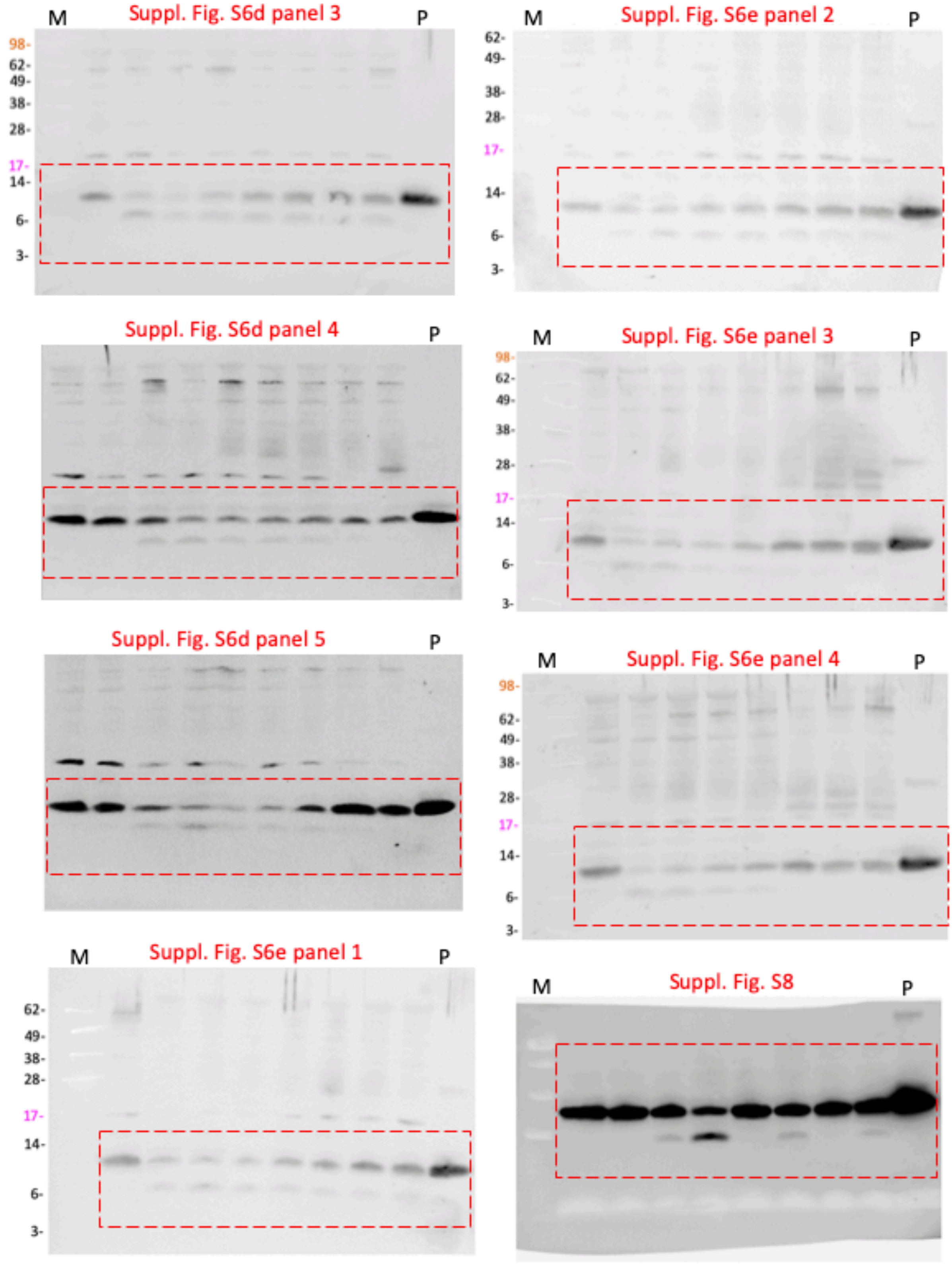

Fig. S9. *(continued)*

Table S1. *D. deserti* proteins with zinc/cysteine sites or putative zinc/cysteine sites.

The list of proteins was obtained after searching *D. deserti* proteins in the UniProt database with keywords zinc, Znf and zf (zinc finger), and by analyzing all *D. deserti* proteins for the presence of the motif CXXCXXC and motif pairs CXXC + CXXC, CXXC + CXC, CXC + CXXC, CXXC + CXXXC, CXXXC + CXXC.

Then all identified proteins were verified for the presence of predicted/annotated Zn/Cys sites (also taking in account data for homologs in other species).

Label	Gene	Product	COG class	eggNOG class	Length	Cys	motif(s)	Zn/Cys	comment
Deide_00340	queC	7-cyano-7-deazaguanine synthase	R	S	237	6	CX(7)CXXCXXC	Zn	Binds 1 zinc ion to: CXXXXXXXCXXCXXC
Deide_00480	priA	Probable primosomal protein N' (ATP-dependent helicase PriA)	L	L	857	13	4 times CXXC; CXXXC	Zn	two C4-type zinc fingers
Deide_01140		Thioredoxin 2	OC	O	138	6	twiceCXXC + CXXC	Zn	zinc finger (Nter two CXXC pairs); third CXXC is redox-active site of thioredoxin
Deide_01220		Putative Carbonic anhydrase	P	P	243	2	CXD + HXXC	Zn	Zn binding to CXD + HXXC
Deide_01330	rpmF	50S ribosomal protein L32	J	J	60	4	twiceCXXC	Zn	
Deide_01610	dnaX	DNA polymerase III subunit gamma/tau	L	L	762	5	CX(7)CXXCXXC	Zn	In Bs & Ec CX(8)CXXCXXC; Zn bound to these 4 Cys in E. coli crystal structures
Deide_01710		acetyl-coenzyme A carboxylase carboxyl transferase subunit beta	I	I	296	4	twiceCXXC	Zn	C4-type zinc finger
Deide_01810		zf-RING_7 domain-containing protein	R	S	240	4	twiceCXXC	Zn	Zf-RING_7; C4-type zinc ribbon domain
Deide_02040		DEAD/DEAH box helicase-like protein	R, L	L	880	10	twiceCXXC; CCX(5)CXXCX(5)C	Zn	PF09369 DUF1998
Deide_02250	ribAB	Riboflavin biosynthesis protein RibBA	H	H	406	6	C + CXC	Zn	Zinc; catalytic
Deide_02270	ribD	Riboflavin biosynthesis protein RibD	H	H	368	3	H + C + C	Zn	Zinc; catalytic
Deide_02700	folE	GTP cyclohydrolase 1	H	H	205	2	CXXH + C	Zn	
Deide_03550	adh	Putative Alcohol dehydrogenase (ADH)	R	C	347	8	C + H + C; CXXCXXCX(7)C	Zn	Binds 2 Zn2+ ions (one catalytic, one not) per subunit. Structural Zn to CXXCXXCXXXXXXXC
Deide_03610	rsgA	Small ribosomal subunit biogenesis GTPase RsgA	R	G	330	3	CXXXXCXHXXXXXC	Zn	Binds 1 zinc ion, to CXXXXCXHXXXXXC
Deide_04740	nrdR	Transcriptional repressor NrdR	K	K	148	4	twiceCXXC	Zn	zinc finger
Deide_04900	dnaG	DNA primase	L	L	572	4	CXXH + CXXC	Zn	DNA Primase, CHC2-type zinc finger
Deide_05880	proS	prolyl-tRNA synthetase	J	J	499	5	CXXXXC + CXXC	Zn	Thermus homolog with structural Cys/Zn
Deide_06020	rpoC	DNA-directed RNA polymerase subunit beta'	K	K	1541	11	CXC + CXXC; CX(6)CXXC	Zn	Binds 2 zinc ions per subunit; Zn1: Cys57-Cys59-Cys72-Cys75; Zn2: Cys1123-Cys1204-Cys1211-Cys1214
Deide_06260	hslO	heat shock protein 33 (HSP33) (33 kDa chaperonin)	O	O	302	4	CXXC + CXC	Zn	
Deide_06340	recR	Recombination protein RecR	L	L	198	5	twiceCXXC	Zn	C4-type zinc finger
Deide_06410	thrS	Threonine--tRNA ligase	J	J	649	4	C + H + H	Zn	binds Zn to C, H, H
Deide_06510		ATP-dependent DNA helicase	L	L	1683	18	CXXXCX(4)CC; CX(15)CXXCXXC	Zn	has CX(15)CXXCXXC similar to CX(16)CXXCXXC in RecQ; RecQ_Zn_bind
Deide_06660	tdk	Thymidine kinase	F	F	202	4	CXXC + CXXH	Zn	
Deide_07570	tdh	L-threonine 3-dehydrogenase	ER	E	344	5	C + HE; CXXCXXCX(7)C	Zn	Binds 2 Zn2+ ions (one catalytic, one not). Structural Zn to CXXCXXCXXXXXXXC
Deide_07590		chaperone protein DnaJ	O	O	373	8	4 times CXXC	Zn	CR-type zinc finger
Deide_08980		Putative DNA/RNA helicase, SNF2 family	KL	L	1132	5	CXC + CXH	Zn	Zinc finger, SWIM-type
Deide_09052	tilS	tRNA(Ile)-lysidine synthase	D, FJ	F	533	3	H + CXXC	Zn	catalytic
Deide_10322		SEC-C motif-containing protein	S	S	126	4	CXC + CC	Zn	Zn found in a crystal structure
Deide_11120		Methionine synthase	E	E	1229	6	C + CC	Zn	Binds 1 zinc ion to C + CC
Deide_11320	recQ	ATP-dependent DNA helicase RecQ	L	L	731	8	CX(16)CXXCXXC	Zn	IPR032284 RecQ_Zn-bd; PF16124 RecQ_Zn_bind
Deide_11761	gluQ	Glutamyl-Q tRNA(Asp) synthetase	J	J	294	5	CXC + YXXXC	Zn	Binds 1 zinc ion per subunit, to CXC + YXXXC
Deide_11961		Cytidine deaminase	F	F	146	4	CXE + CXXC	Zn	Zn to CXE + CXXC
Deide_12290	ligA2	DNA ligase 2	L	L	686	4	CXXC + CXXXXC	Zn	Znf_DNAligase_C4
Deide_12480		Delta-aminolevulinic acid dehydratase	H	H	333	4	CXC + C	Zn	Zn binding to CXC + C (catalytic)
Deide_12660	radA	DNA repair protein RadA	O	O	449	5	twiceCXXC	Zn	C4-type zinc finger
Deide_12760	uvrA1	UvrABC system protein A (UvrA protein)	L	L	1004	14	5 times CXXC	Zn	two C4-type zinc fingers

Deide_13450		zinc finger motif protein		S	113	8	4 times CXXC	Zn	found for some homologs: Zinc finger, DPH-type; PF13717 zinc_ribbon_4
Deide_13810	recO	DNA repair protein RecO	L	L	247	4	twiceCXXC	Zn	IPR042242 RecO_ZnD
Deide_14130		metallo-beta-lactamase family protein	R	S	263	4	CXCXXC	Zn	Zn in crystal structure of E. coli homolog
Deide_14260		hypothetical protein	R	S	291	5	twiceCXXC	Zn	COG5512: Predicted nucleic acid-binding protein, contains Zn-ribbon domain; gene is downstream of recF
Deide_16041		ferric uptake regulator (FUR family)	P	K	127	5	twiceCXXC	Zn	structural
Deide_16240	mutM fpg	Formamidopyrimidine-DNA glycosylase (Fapy-DNA glycosylase)	L	L	276	5	twiceCXXC	Zn	zf-FPG_IleRS
Deide_16410	cysS	Cysteine--tRNA ligase	J	J	491	2	C + C + HXXXE	Zn	Zn bound to C, C, H, E
Deide_17280		S-(hydroxymethyl)glutathione dehydrogenase (Class III alcohol dehydrogenase)	ER	C	400	11	CXXCXXCX(7)C; CXXXC; CXXC	Zn	Cys90-Gly91-Cys93-Cys96-Cys104: structural zinc binding site; Cys38 and 3 other aa: catalytic zinc binding site
Deide_18730	ddrT	Putative SWIM zinc finger domain protein		S	433	7	CXC + CXH	Zn	Zinc finger, SWIM-type
Deide_18820	rpsZ	30S ribosomal protein S14 type Z	J	J	61	4	twiceCXXC	Zn	
Deide_19270	alaS	Alanine--tRNA ligase	J	J	891	5	CXXC; HXXXH + CXXXH	Zn	Zn bound to HXXXH + CXXXH
Deide_19480		ferric uptake regulator (FUR family)	P	K	133	4	twiceCXXC	Zn	structural
Deide_19510		HNH endonuclease	V	V	188	4	twiceCXXC	Zn	Zn found in Gmet_0936 structure
Deide_19580	clpX	ATP-dependent protease ATP-binding subunit ClpX	O	O	401	4	twiceCXXC	Zn	zf-C4_ClpX (ClpX C4-type zinc finger)
Deide_19610		Type II secretion system protein E, Type IV pilus assembly protein	NU	U	894	4	twiceCXXC	Zn	PF00437: The cytoplasmic T2S E ATPase is a Zn-containing protein...; Rule et al 2016 Microbiologyopen 5(5):870-882
Deide_1p00290	ligA1	DNA ligase 1	L	L	686	6	CXXC + CXXXXC	Zn	Znf_DNAligase_C4
Deide_1p01280		ATP-dependent DNA helicase RecQ-like	L	L	548	11	CX(16)CXXCXXC	Zn	PF16124 RecQ_Zn_bind; C4-type zinc finger; other Cys not conserved
Deide_20612	tgt	Queuine tRNA-ribosyltransferase	J	J	385	6	CXCXXC + H	Zn	Binds 1 zinc ion, to CXCXXC + H
Deide_21710		putative DNA polymerase III subunit delta'	L	L	318	4	CX(10)CXXCXXC	Zn	Motif also in other Deinos. In E. coli HolB: CX(8)CXXCXXC. Zn bound to these 4 Cys in E. coli crystal structure.
Deide_22560		Putative Cytidine/deoxycytidylate deaminase, zinc-binding region	FJ	F	221	5	CXXC	Zn	Nucleoside/Zn binding site includes CXXC
Deide_23030		Putative dCMP deaminase (Deoxycytidylate deaminase)	F	F	142	3	H + CXXC	Zn	Zn binding to H + CXXC (catalytic)
Deide_2p00340		Ferric uptake regulator (FUR family)	P	P	142	4	twiceCXXC	Zn	structural
Deide_2p02060	uvrA2	UvrABC system protein A (UvrA protein)	L	L	846	8	4 times CXXC	Zn	zinc finger; 2 zinc ions
Deide_3p00853		Putative homocysteine S-methyltransferase	E	E	314	7	C + CC	Zn	Zn binding to C + CC
Deide_3p02130		Putative sorbitol dehydrogenase (Alcohol dehydrogenase)	ER	C	364	7	CXXCXXCX(7)C	Zn	catalytic Zn binding site: CXS + HE + E; structural Zn site: CXXCXXCXXXXXXXC.
Deide_3p02772		zf_CopZ domain-containing protein		P	163	12	several	Zn	Nter: PF18423 zf_CopZ (zinc binding domain found in copper chaperone CopZ proteins; CXXC + CXXXXC); Cter: IPR041854 BFD-like_2Fe2S-bd_dom_sf
Deide_01281	rpmB	ribosomal protein L28	J	S	73	4	twiceCXXC	Zn?	
Deide_04200		hypothetical protein		S	262	5	twiceCXXC	Zn?	
Deide_04721	ddrS	conserved protein of unknown function			74	4	twiceCXXC	Zn?	
Deide_10375		hypothetical protein	K	K	98	5	CXXC + CX(5)C	Zn?	DUF448 domain-containing protein; Some member sequences retain zinc-binding residues.
Deide_02401		hypothetical protein	R	S	111	9	CXXCXXCC; CXC	Zn or Fe	CxxCxxCC (PF03692): Putative zinc- or iron-chelating domain
Deide_11910		Uncharacterized protein	R	S	249	8	CXXXCXXCC	Zn or Fe	Putative zinc- or iron-chelating domain
Deide_02560	cadA	Lead, cadmium, zinc and mercury-transporting ATPase	P	P	729	4	CXXC + CXC	?	1 HMA domain (heavy metal-associated); for Cu, Co, Cd, Zn...?
Deide_02570		transcriptional repressor, ArsR/SmtB family	K	K	125	5	CXXXC + CXC	?	downstream cadA homolog; some with reported binding Zn to Cys and other aa, others not. Also reported Cd binding

Deide_04590	paaD	phenylacetic acid degradation protein	R	Q	164	6	CXXC + CX(5)CXXC	?	FeS_assembly_P domain (PF01883) at Nter, but 2 CXXC motifs are at Cter
Deide_04660		ribonucleoside-diphosphate reductase	F	F	996	13	CXXC + CXXCXXCX(4)C	?	
Deide_05260		conserved protein of unknown function			62	4	CXXXXC	?	DUF1540 domain-containing protein; These proteins have four conserved cysteines, which is suggestive of a metal binding function. This domain may be found on its own or duplicated in the proteins.
Deide_05371		putative competence protein ComF	R	S	212	7	twiceCXXC	?	
Deide_08531		hypothetical protein	V	V	109	4	twiceCXXC	?	homologous to Deide_16091
Deide_08850		putative protease, Peptidase U32 family	O	O	854	11	twice CXXXC; CXXC	?	
Deide_11020		hypothetical protein (RNHCP domain-containing protein)		S	119	7	3 times CXXC	?	
Deide_11660	ileS	Isoleucine--tRNA ligase	J	J	1066	5	CXXC; CXXH	?	T. thermophilus homolog binds 2 Zn with 8 Cys, but not all Cys conserved in Deide_11660
Deide_13081		hypothetical protein			183	6	CXXC + CXC	?	
Deide_13820		hypothetical protein			136	4	CXXC + CX(5)C	?	well conserved in Deino, incl. Cys
Deide_14170	leuS	Leucine--tRNA ligase	J	J	831	7	twiceCXXC; CXXH	?	
Deide_16091		hypothetical protein	V	V	110	4	twiceCXXC	?	homologous to Deide_08531
Deide_16150		UPF0176 protein Deide_16150 (Rhodanese domain-containing protein)	R	J	304	9	twiceCXXC; CX(5)C	?	
Deide_17805		hypothetical protein	S	S	61	4	CXXC + CXXXC	?	gene upstream of uvsE
Deide_19425		conserved cysteine-rich protein of unknown function	C	C	113	18	several	?	DUF326; homologous to Deide_21851; copper storage?
Deide_1p00170		hypothetical protein		S	734	10	twiceCXXC; CXXH; CC	?	only few homologs but CXXC, CXXH and CC conserved
Deide_1p01700		copper-exporting ATPase; membrane protein	P	P	835	6	twiceCXXC; CXC	?	2 HMA domains (heavy metal-associated); for Cu, Co, Cd, Zn…?
Deide_20550		hypothetical protein	R	S	200	6	CXXC; CXXXCXXCX(8)C	?	PF02620 YceD; COG1399: Predicted metal-binding, possibly nucleic acid-binding protein
Deide_20690		hypothetical protein		S	83	4	CXXC	?	well conserved in Deino, incl. Cys
Deide_21130		Prepilin peptidase	NOU	N	381	5	twiceCXXC	?	in cytoplasmic domain
Deide_21851		hypothetical protein	C	S	152	18	several	?	homologous to Deide_19425; copper storage?
Deide_22810		conserved protein of unknown function	R	S	88	6	CXC	?	most Cys (not first C of CXC) conserved in Deino
Deide_2p00110		hypothetical protein		S	179	6	CX(5)C + CX(5)CC	?	well conserved in several Deino, incl. Cys
Deide_2p00780		hypothetical protein		S	1070	5	CXXC; CXXXC; CXXH	?	
Deide_3p00552		hypothetical protein			110	4	CXXC	?	well conserved in few Deino, incl. Cys
Deide_3p01430		hypothetical protein			245	4	CX(17)CXXCXXC	?	No homologs

Table S2. Bacterial strains.

**Strain**	**Genotype or relevant characteristics**	**Source or reference**
Escherichia coli		
BL21 (AI)	F- *ompT* *hsdS*$_B$ (r_B-m_B-) *gal dcm araB::*T7RNAP-*tetA*	Invitrogen
TOP10	F- *mcr*A Δ(*mrr-hsd*RMS-*mcr*BC) Φ80*lac*ZΔM15 Δ*lac*X74 *rec*A1 *ara*D139 Δ(*ara,leu*)7697 *gal*U *gal*K *rps*L (Str^R) *end*A1 *nup*G	Invitrogen
Deinococcus deserti		
RD19	As wild-type strain VCD115 but streptomycin resistant (Str^R)	[5]
RD42	As RD19 but Δ*irrE*Ω*kan*	[5]
RD63	As RD19 but Δ*ddrS*Ω*kan*	This work
RM1	As RD19 but Δ*bshA*Ω*kan*	This work
RM2	As RD19 but Δ*bshC*Ω*kan*	This work
Deinococcus radiodurans		
DSM 20539	Type strain	Laboratory stock

Table S3. Plasmids.

Plasmid	Description	Source or reference
pCR4Blunt-TOPO	Cloning vector, Amp^R, Kan^R	Invitrogen
pET-TEV	pET28a derivative, expression vector for *E. coli*, Kan^R	[6]
p12714	*D. deserti irrE* in pET-TEV, Kan^R	[5]
pML6	As p12714 but encoding IrrE lacking its GAF-like domain (IrrEΔGAF)	This work
pET22ddrO	*D. deserti* $ddrO_C$ in pET22b, Amp^R	[1]
pRD48	*D. deserti irrE* and its promoter in pCR4Blunt-TOPO	[5]
pI3	Shuttle vector that replicates in *E. coli*, *D. radiodurans* and *D. deserti*; Amp^R in *E. coli*, Cm^R in *Deinococcus*	[7,8]
pRD51	*D. deserti irrE* and its promoter in pI3	[5]
pI3-irrE-M18V	As pRD51 but encoding IrrE-M18V	This work
pI3-irrE-M243L	As pRD51 but encoding IrrE-M243L	This work
pI3-irrE-C116A	As pRD51 but encoding IrrE-C116A	This work
pI3-irrEΔGAF	As pRD51 but encoding IrrEΔGAF	This work
pI3-irrEΔGAF-SPA	As pRD51 but encoding IrrEΔGAF fused to SPA-tag	This work
p14001	Source of SPA-tag	[9]

Table S4. Primers

Primer	Primer sequence	Mutation or amplified fragment
IrrE_M243L_for	TTCGGGGCGCCGCCTGCCAGCCTACGT	ATG -> CTG
IrrE_M243L_rev	ACGTAGGCTGGCAGGCGGCGCCCCGAA	
IrrE_M18V_for	CAGCAAAAGCTCGCGTCCGTGAGCTCGCGG	ATG -> GTC
IrrE_M18V_rev	CCGCGAGCTCACGGACGCGAGCTTTTGCTG	
C116A_for	GATTGAGACGCTGGCTAACGTGGGCGC	TGT -> GCT
C116A_rev	GCGCCCACGTTAGCCAGCGTCTCAATC	
FV45	TTAA**CATATG**ACGGATCCCGCTCCTCCG	*irrE*ΔGAF flanked by NdeI and EcoRI sites, for cloning in pET-TEV
D2_EcoRI_Rv	TAA**GAATTC**TCACGCCGTACGTTCAGCCAG	
irrE-XbaFW2	GAA**TCTAGA**GCTCAGCGGCAGTAAACC	*irrE* promoter region + *irrE*ΔGAF flanked by restriction sites XbaI and HindIII, for cloning in pI3
D2_Hd3_Rv	TAA**AAGCTT**TCACGCCGTACGTTCAGCCAG	
irrE-XbaFW2	GAA**TCTAGA**GCTCAGCGGCAGTAAACC	*irrE* promoter region + *irrE*ΔGAF flanked by XbaI site and the beginning of SPA-tag[a]
D2_SPA_Rv	CTTCTCTTTTCCATGGACGCCGTACGTTCAGCCAG	
SPA_D2_Fw	TGGCTGAACGTACGGCGTCCATGGAAAAGAGAAGATG	SPA-tag flanked by the end of IrrE-HTH domain and restriction site HindIII[b]
SPA_Hd3_Rv	GAG**AAGCTT**CTACTTGTCATCGTCATCCTTGT	

[a]for fusion PCR with SPA-tag (and subsequent cloning in pI3)

[a]for fusion PCR with region containing *irrE* promoter and *irrE*ΔGAF (and subsequent cloning in pI3)

References in Supplemental Figures and Tables

1. Ludanyi, M. *et al.* Radiation response in *Deinococcus deserti*: IrrE is a metalloprotease that cleaves repressor protein DdrO. *Mol. Microbiol.* **94,** 434–449 (2014).
2. Blanchard, L. *et al.* Conservation and diversity of the IrrE/DdrO-controlled radiation response in radiation-resistant *Deinococcus* bacteria. *Microbiologyopen* **6,** e477 (2017).
3. de Groot, A. *et al.* Crystal structure of the transcriptional repressor DdrO: insight into the metalloprotease/repressor-controlled radiation response in *Deinococcus*. *Nucleic Acids Res.* **47,** 11403–11417 (2019).
4. Winn, M. D. *et al.* Overview of the CCP4 suite and current developments. *Acta Crystallogr. D Biol. Crystallogr.* **67,** 235–242 (2011).
5. Vujicic-Zagar, A. *et al.* Crystal structure of the IrrE protein, a central regulator of DNA damage repair in Deinococcaceae. *J. Mol. Biol.* **386,** 704–716 (2009).
6. Houben, K., Marion, D., Tarbouriech, N., Ruigrok, R. W. & Blanchard, L. Interaction of the C-terminal domains of sendai virus N and P proteins: comparison of polymerase-nucleocapsid interactions within the paramyxovirus family. *J. Virol.* **81,** 6807–6816 (2007).
7. Masters, C. I. & Minton, K. W. Promoter probe and shuttle plasmids for *Deinococcus radiodurans*. *Plasmid* **28,** 258–261 (1992).
8. Meima, R. & Lidstrom, M. E. Characterization of the minimal replicon of a cryptic *Deinococcus radiodurans* SARK plasmid and development of versatile *Escherichia coli-D. radiodurans* shuttle vectors. *Appl. Environ. Microbiol.* **66,** 3856–3867 (2000).
9. Bouthier de la Tour, C. *et al.* The abundant and essential HU proteins in *Deinococcus deserti* and *Deinococcus radiodurans* are translated from leaderless mRNA. *Microbiology* **161,** 2410–2422 (2015).
